# Factors Controlling Persistent Needle Crystal Growth: The Importance of Dominant One-Dimensional Secondary Bonding, Stacked Structures, and van der Waals Contact

**DOI:** 10.1021/acs.cgd.1c00217

**Published:** 2021-05-21

**Authors:** Francesco Civati, Ciaran O’Malley, Andrea Erxleben, Patrick McArdle

**Affiliations:** † School of Chemistry, National University of Ireland, Galway H91TK33, Ireland; ‡ Synthesis and Solid State Pharmaceutical Centre (SSPC), Limerick V94T9PX, Ireland

## Abstract

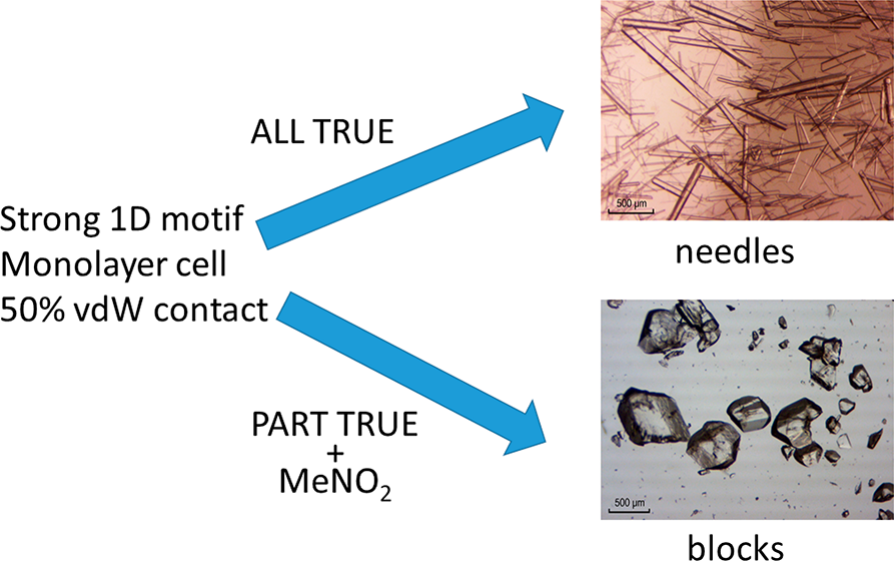

Needle crystals can cause filtering and handling problems in industrial settings, and the factors leading to a needle crystal morphology have been investigated. The crystal growth of the amide and methyl, ethyl, isopropyl, and t-butyl esters of diflunisal have been examined, and needle growth has been observed for all except the t-butyl ester. Their crystal structures show that the t-butyl ester is the only structure that does not contain molecular stacking. A second polymorph of a persistent needle forming phenylsulfonamide with a block like habit has been isolated. The structure analysis has been extended to known needle forming systems from the literature. The intermolecular interactions in needle forming structures have been analyzed using the PIXEL program, and the properties driving needle crystal growth were found to include a 1D motif with interaction energy greater than −30 kJ/mol, at least 50% vdW contact between the motif neighbors, and a filled unit cell which is a monolayer. Crystal structures are classified into persistent and controllable needle formers. Needle growth in the latter class can be controlled by choice of solvent. The factors shown here to be drivers of needle growth will help in the design of processes for the production of less problematic crystal products.

## Abstract 2

Crystal structures can be classified as persistent and controllable needle formers. Persistent needle formers have a consistent set of properties, while systems which do not have these properties have morphologies which can be controlled by solvent choice.

## Introduction

Crystal morphology can have a major impact on the isolation and downstream processing of active pharmaceutical ingredients (APIs). Needle crystal morphology can be particularly problematic in that needles are difficult to filter, tend to clog equipment, and break easily creating unwanted fines.^1−3^ The factors controlling crystal morphology, including needle crystal morphology, have often been examined using the Bravais–Friedel–Donnay–Harker method, BFDH,^4^ periodic bond chain, PBC, analyses^5,6^ and slice attachment energies.^7^ There have also been some theoretical simulations of crystal growth mechanisms including Monte Carlo methods applied to idealized growth units^8^ and molecular dynamics simulations applied to both crystal growth and dissolution.^9^ The computationally demanding molecular dynamics simulations have thus far only been applied to the simplest systems with few degrees of freedom. A study of needle growth using PBC analysis combined with crystal growth mechanisms has suggested that systems may be divided into absolute and conditional needle formers.^6,10^ In this paper we examine the structures of compounds from the literature and new systems to determine the range of factors which influence needle growth including the strength of the intermolecular forces, molecular shape, and stacking motifs. We will attempt to classify systems which can crystallize as needles into persistent and controllable classes. The aim of the paper is to provide criteria derived from crystal structures which will indicate when it is worthwhile to try to control needle growth by adjusting crystallization conditions. To this end we will first briefly comment on needle growth and morphology prediction and then discuss the crystal structures and the morphology of diflunisal derivatives, of 2′-hydroxy[1,1′-bicyclohexyl]-1-carbonitrile and of a new polymorph and solvate of 4-hydroxy-*N*-phenylbenzenesulfonamide, and finally analyze additional examples of needle formers from the literature.

## Results and Discussion

### Unique Properties of Needle Crystals

Needle crystals are observed for crystal growth from the gas phase,^11,12^ from solution,^13^ and from melts.^14^ Needle growth is also reversible and needle crystals have been observed to get shorter faster than they get thinner for both needle sublimation^15^ and needle dissolution.^16^ During crystal growth needles have been observed to have smooth side faces and needle tips which have a rough or rounded appearance.^11,13,17,18^ Since needle crystals with high aspect ratios would be expected to have higher energies than crystals with a more equant thermodynamically favored shape it should be possible to observe a reduction of needle crystal aspect ratios in solution under equilibrium conditions. This has in fact been observed for isonicotinohydrazide and diflunisal needles in ethanol at ambient temperature under high liquid shear low mechanical attrition conditions.^16^

### Crystal Growth Mechanisms

**Figure 1:**
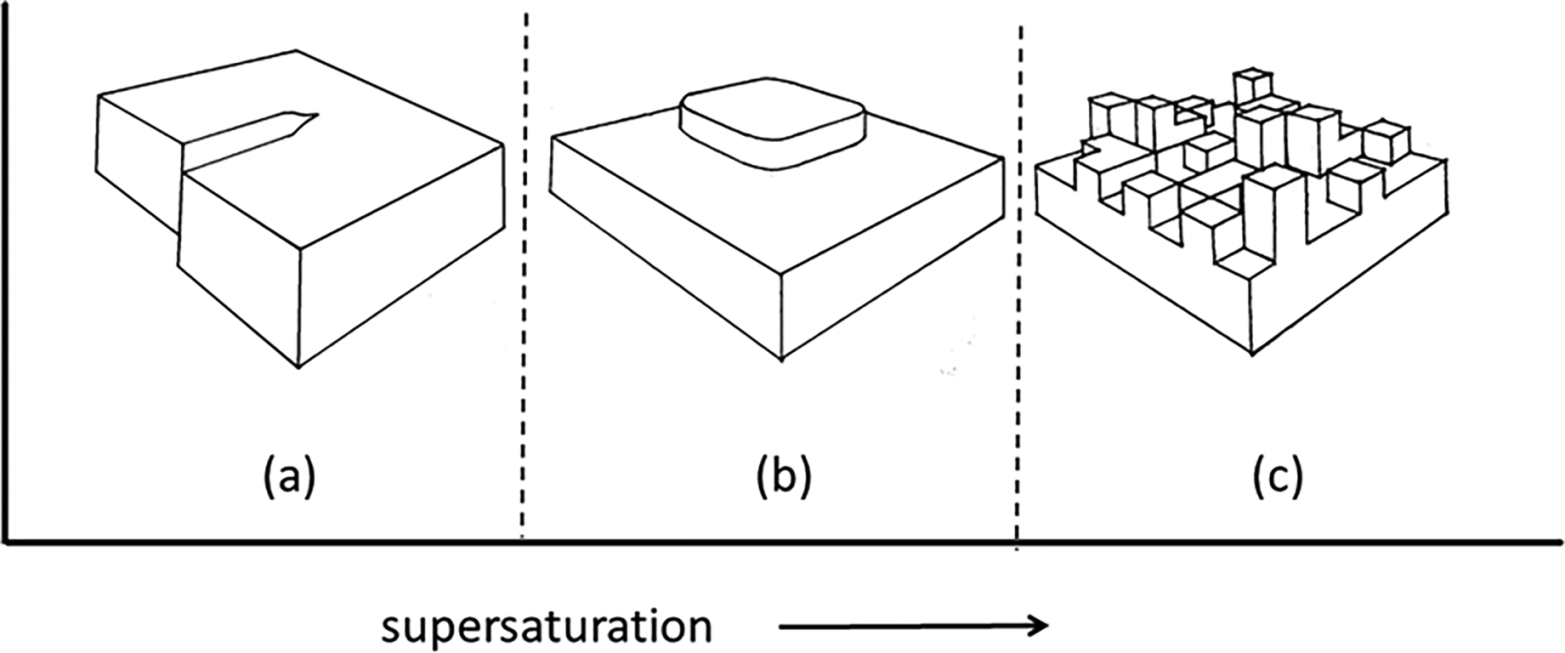
(a) Screw dislocation providing a constant nucleation source for spiral growth at low supersaturation, (b) 2D nucleation at moderate supersaturation, and (c) rough growth at high supersaturation.

Just as crystal nucleation requires the formation of a critical size cluster of molecules^19^ the growth of a new layer on a smooth crystal face requires nucleation. Calculations have shown that at low supersaturation the rate of growth of a smooth crystal face should be close to zero.^20^ However, if dislocations are present in the crystal structure such as screw dislocations then smooth spiral growth at low supersaturation becomes a favorable process.^20^ This Burton Cabrera Frank, BCF, mechanism can lead to layer by layer growth with the dislocation providing a constant source of nucleation for new layers, Figure 1a. At higher supersaturation levels it is believed that two-dimensional nucleation is possible on a smooth crystal face and layer by layer growth is again possible, Figure 1b.^21^ At even higher supersaturation a transition to multimolecular layer or rough growth is possible, Figure 1c.^22^

For example, sucrose gives block like crystals with smooth crystal faces when grown from aqueous solution with 2.2% relative supersaturation, but at 5.1% supersaturation all faces show rough growth without a change in crystal habit.^23^ In contrast to what is observed for crystals with a block like habit, the form I polymorph of *p*-aminobenzoic acid, PABA, grows as needles from ethanol with a 2D birth and spread mechanism on the needle side faces leading to smooth growth and a rough growth mechanism, even at low supersaturations, on the needle capping faces.^24^ Thus, needle growth differs from “normal” crystal growth in that growth in the direction of the needle axis is rough growth while the needle side faces always have smooth growth. It has been estimated that the energy required for the generation of a 2D nucleus on the needle tip faces of needle forming β-triacylglycerol is close to zero and this is why its needle tips always grow rough.^25^

It has also been shown that for needle crystal growth from the gas phase the aspect ratio is inversely related to the crystallization driving force for benzoic acid and 1,4-naphthoquinone^12^ and for β-phthalocyanine using two different experimental setups.^11,12^ Any chemical process which shows less discrimination at higher reaction rates is an example of the reactivity selectivity principle, RSP, which was once believed to have wide application in chemistry, but by the 1970s, it was believed that there were many exceptions to RSP.^26,27^ More recently and after much detailed examination it has been suggested that as far as most chemical reactions are concerned the idea is a myth.^28^ Nevertheless a significant number of reactions still follow the principle, and a reduction in the activation energy is related to a reduction in selectivity.^29^ Many enantioselective catalysts show higher selectivity at lower temperatures and lower reaction rates.^30^ The noncovalent interactions which control the approach of substrates in chiral catalysis^31^ are similar to the interactions involved in the addition of a molecule in the correct orientation to a growing crystal and lead to their adherence to RSP.

### Crystal Morphology Predictions based on BFDH and Slice Attachment Energy

**Figure 2:**
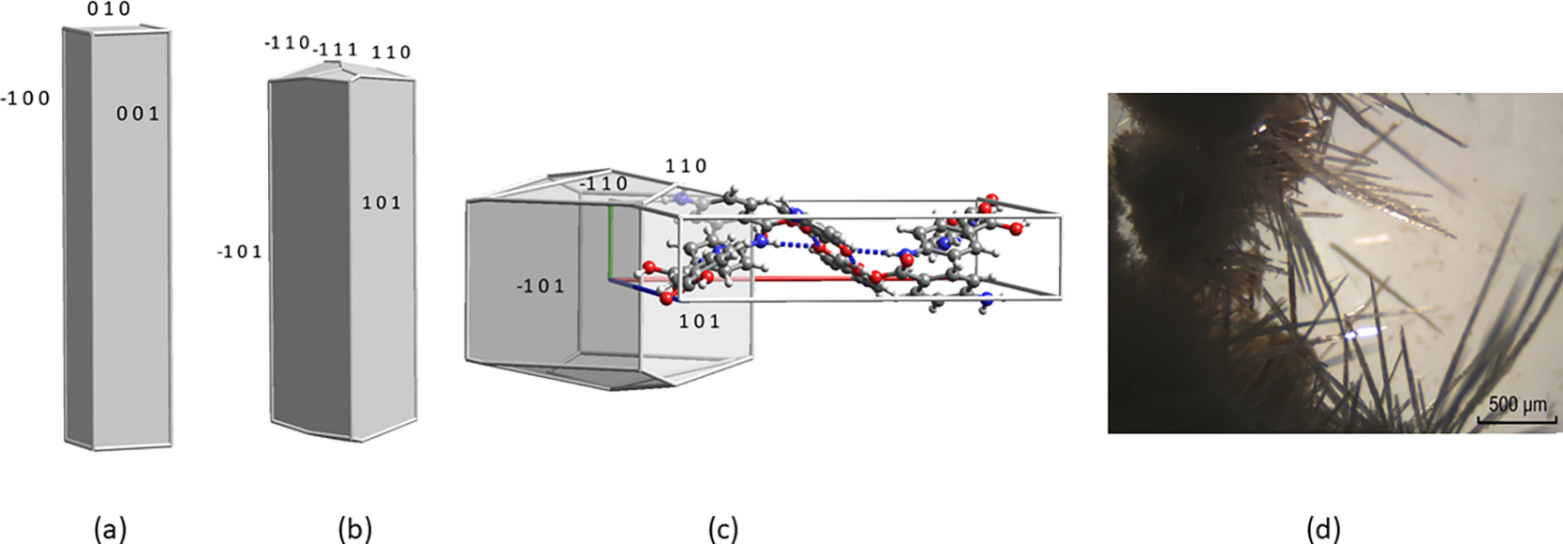
Crystal morphology of PABA form I predicted by BFDH (a) simplified and ignoring space group extinctions, (b) including space group extinctions, (c) SAE morphology calculated using the Habit program, and (d) PABA form I crystals grown by sublimation.

Methods for the prediction of crystal morphology have developed from the early work on the BFDH method^4^ through the slice attachment energy model, SAE,^7,32,33^ and modifications to attachment energy which try to include the effect of solvent on solution grown crystals.^34^ These methods are based on thermodynamic considerations alone in that crystal growth is assumed to be driven by the energy released when molecules are added to the growing crystal and mechanistic factors are usually ignored. It is often stressed that the BFDH method is based on the unit cell dimensions alone and that it ignores the unit cell contents.^35^ Clearly the attempts to improve BFDH morphology prediction using slice attachment energies by calculation of intermolecular energies have a sound logical basis; nevertheless, BFDH predictions of crystal morphology are still widely used. A Google Scholar search using the search term “BFDH” for the period 2000–2020 gave 1810 hits. It is important to understand why the BFDH method has such enduring appeal. The BFDH law states that the morphological importance of a crystal face is directly proportional to its *d*-spacing and extinctions due to translational symmetry elements must be taken into account. A parallelepiped shape is normally assumed.^4^ The morphologically important faces are the slowest growing faces, and thus, the rate of growth of a crystal face is inversely proportional to its *d*-spacing and directly proportional to face area. This is illustrated using calculated morphologies for PABA form I in Figure 2.

In Figure 2 the calculated morphologies all have lower aspect ratios than the needle like crystals grown by sublimation. The *b* unit cell face has the largest area, Figure 2c and Table 1, and the (010) crystal face would be expected to have the fastest growth rate. This is why it is invariably the case, as is discussed below, that needle crystals grow in the direction of the shortest crystal axis. The BFDH rule gives reasonable results in many cases because in molecular crystals the larger the facial area the greater the number of intermolecular interactions on that face and the greater the energy released by growth in that direction. To a considerable extent specific directional effects tend to average out. Using the PABA form I structure it is possible to count the number of accessible atoms in a unit cell face using a probe moving on a 0.1 Å grid.^36^ The numbers of atoms encountered by the probe, and the areas of the (100), (010), and (001) faces are compared and scaled with errors in Table 1.

**Table 1:** PABA Form I Face Areas and Numbers of Atoms on Faces Encountered by a Probe

face	area/Å^2^	atoms	atom number scaled to area	HB donors	HB donors scaled to area	HB acceptors	HB acceptors scaled to area
(100)	69.296	20	70.29	3	89.38	4	71.2
(010)	343.824	98	344.4	10	297.92	19	338.20
(001)	69.273	14	49.2	5	148.96	5	89.0
error			0.033		0.152		0.033

Despite the far from spherical shape of PABA form I the numbers of H-bond donors and acceptors encountered on the crystal faces are approximately in proportion to the face area. Thus BFDH by a process of averaging gives a morphology prediction that is based in a general way on the unit cell contents.

### Periodic Bond Chains and Slice Attachment Energies

The Hartman–Perdok theory is based on the concept of periodic bond chains, PBCs.^32^ PBCs are secondary bonding interactions between molecules in the lattice such as H-bonds, dipole–dipole interactions, and vdW interactions which are all termed bonds. Particular importance is attached to bond chains which extend throughout the crystal structure. The usual procedure is to first determine a set of strong bonds and then all PBCs. Finally crystal faces are classified into F faces which have slices containing two types of different PBCs, S type which have one PBC, and K faces which contain none. Using estimated energies for the bonds, the energy released by adding a crystal slice to a particular face can be calculated; its slice attachment energy, SAE, can be calculated, and the rate of growth of that crystal face is then proportional to its SAE. However, if suitable interatomic potential functions are used, SAEs can be calculated without the need for the somewhat subjective PBC analysis, Figure 1c.^7,33^

The observed morphology of PABA form I crystals grown from a range of solvents is needle like with extended growth along the *b* axis, and the needles are often hollow due to rapid growth.^37^ PABA form I also grows as needles from the gas phase^38^ where specific solvent effects are not involved, and as pointed out above, the aspect ratio greatly exceeds the growth rate that would be expected from BFDH, SAE, or indeed any predictions based on thermodynamic considerations alone.

### Substituent Effects on Needle Growth in Diflunisal Derivatives

**Figure 3:**
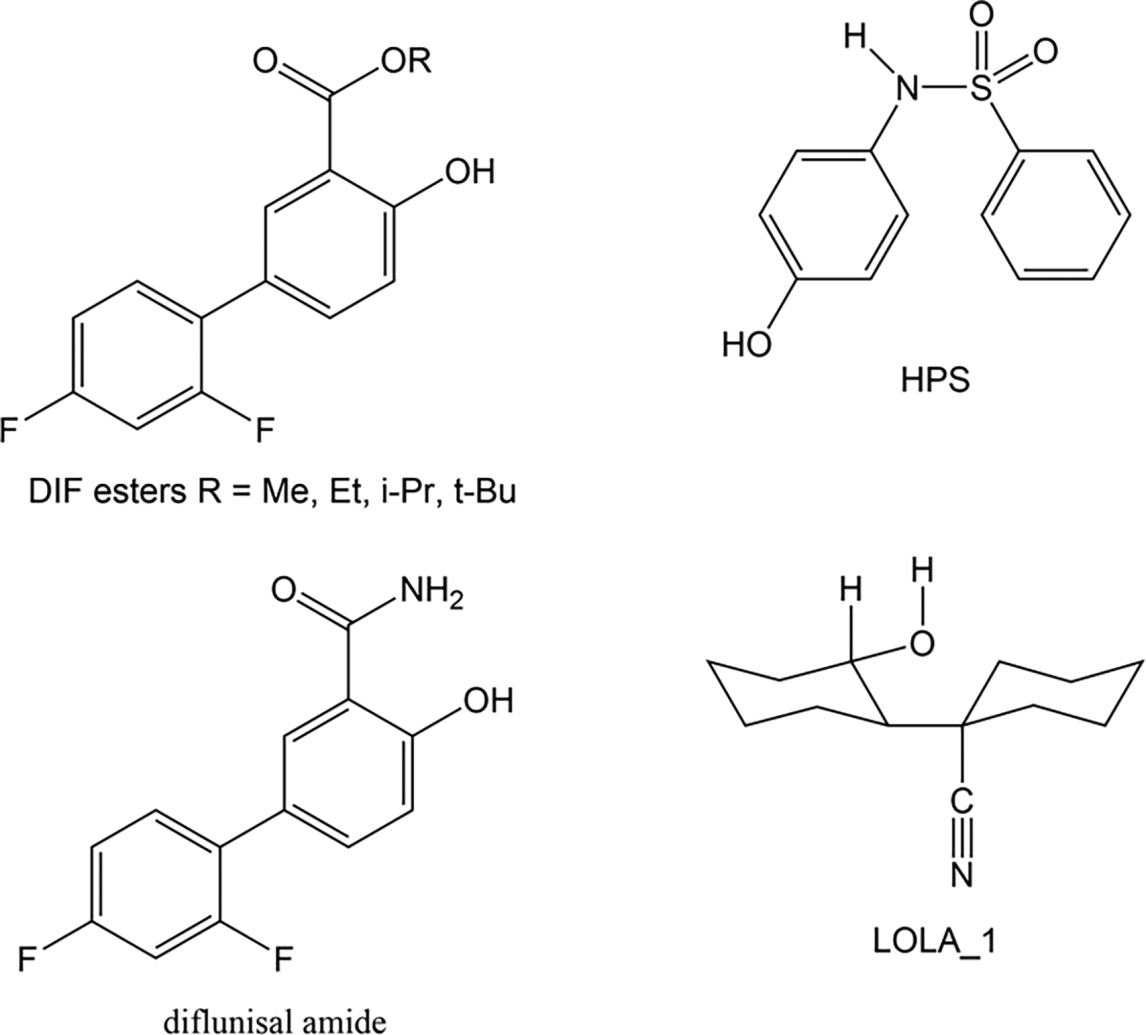
Structural formulas of the compounds used in this study.

5-(2,4-Difluorophenyl)-2-hydroxybenzoic acid or diflunisal (DIF, Figure 3) has four known polymorphs all of which crystallize as needles.^39^ The observation of needle growth in DIF polymorphs and DIF cocrystals has been associated with the presence of molecular stacking in their crystal structures.^40^ The methyl, ethyl, isopropyl, and tertiary butyl esters of DIF and the acetonitrile solvate of diflunisal amide were crystallized, and their crystal structures were determined to test the effect of these substituents on needle growth. The crystal data for the diflunisal esters are in Table S1, and more details of the structures are in the Supporting Information, SI, including data for diflunisal form III.

**Figure 4:**
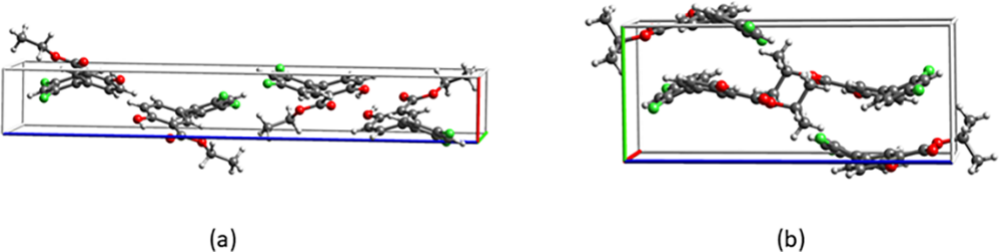
Packed unit cells of (a) the ethyl ester and (b) the isopropyl ester of diflunisal.

The extent to which molecular stacking influences needle growth can be related to the intermolecular energy between stack neighbors calculated by the PIXEL program^41^ and more rapidly estimated by the percent of atoms in a molecule that are in van der Waals (vdW) contact with their stack neighbors.^40^ These figures are given in Table 2 for DIF form III and the diflunisal esters. While there is a small fall in the percent of atoms in vdW contact from DIF form III to the isopropyl ester, the values are all high (>70%) and there is a 50% increase in the interaction energy. Packed unit cells of the ethyl and isopropyl esters are shown in Figure 4.

**Table 2:** Properties and the Persistence of Needle Growth^a^

compound/polymorph	1D motif^b^, direction, and energy/kJ/mol	% atoms in vdW contact	molecular flatness	cell a monolayer	packing index	aligned to unit cell	persistent needle growth	ref
diflunisal form III; FAFWIS02	stack c −30.6	87.5	0.22	yes	72.8	yes	yes	(48)
diflunisal methyl ester	stack a −33.6	82.76	0.41	yes	71.2	yes	yes	this work
diflunisal ethyl ester	stack a −40.7	82.81	0.40	yes	70.0	yes	yes	this work
diflunisal *i*-propyl ester	stack b −45.5	70.0	0.53	no	70.4	yes	yes	this work
diflunisal *t*-butyl ester	none		0.39	no				this work
diflunisal amide solvate	stack b −30.4	83.3	0.48	yes	69.5	yes	yes	this work
HPS1; VUKRAW	stack a −35.1	51.79	0.75	yes	68.0	yes	yes	(44)
HPS2	none		0.83	no	65.8	no	no	this work
HPS aniline solvate	stack a −48.9, −13.2	55.36, 46.43	0.53, 0.09	yes	70.7	yes	yes	this work
HBCN	s-HB c −32.1	3	0.66	no	66.5	yes	no	this work
thymine; THYMIN03	d-HB b −74.8	20	0.29	yes	72.6	yes	no	(49)
succinic acid; SUCACB18	d-HB along [101] −75.2	57, 54, 55	0.25	no	76.6	no	no	(50)
d-mannitol; DMANTL01	t-HB c −99.9	56	0.42	yes	74.2	yes	yes	(51)
aspartame hemihydrate; DAWGOX	HB c −136.3, in stack disp −60	57	0.40	yes	67.1	yes	yes	(52)
aspartame; KETXIR	HB b −98 −127 in stack disp −60	52.55	0.69	yes	68.9	yes	yes	(53)
3-isobutyl-1-methylxanthine; CEWVIJ10	stack a −32.0	61.67	0.48	yes	70.9	yes	yes	(54)
PABA form I; AMBNAC07	stack b −14.2	77.94	0.02	yes	73.5	yes	no	(37)
PABA form V; AMBNAC09	stack b mean −14.3^c^	79.41	0.03	yes	74.0	yes	no	(55)
MNA; MNIANL05	stack c −10.7	46.9	0.01	yes	72.6	yes	no	(56)
NMBA; NMBYAN01	stack a −21.2	43.3	0.13	yes	75.9	yes	no	(57)
β-phthalocyanine; PHTHCY14	stack b −101.5	70.69	0.01	yes	72.7	yes	yes	(58)
lovastatin; CEKBEZ01	stack a −54.8, s-HB b −31.6	42.31	0.72	yes	69.9	yes	no	(59)

a Compounds indicated in the eighth column as persistent needle formers have a stacking interaction energy that is greater than −30 kJ/mol, >50% of their atoms in vdW contact within the stack, and filled unit cells which are monolayers.

b HB = hydrogen bond, s-HB = single hydrogen bond, d-HB = double hydrogen bond, t-HB = triple hydrogen bond, in stack disp = dispersion energy.

c The asymmetric unit contains two molecules that are both stacked.

**Figure 5:**
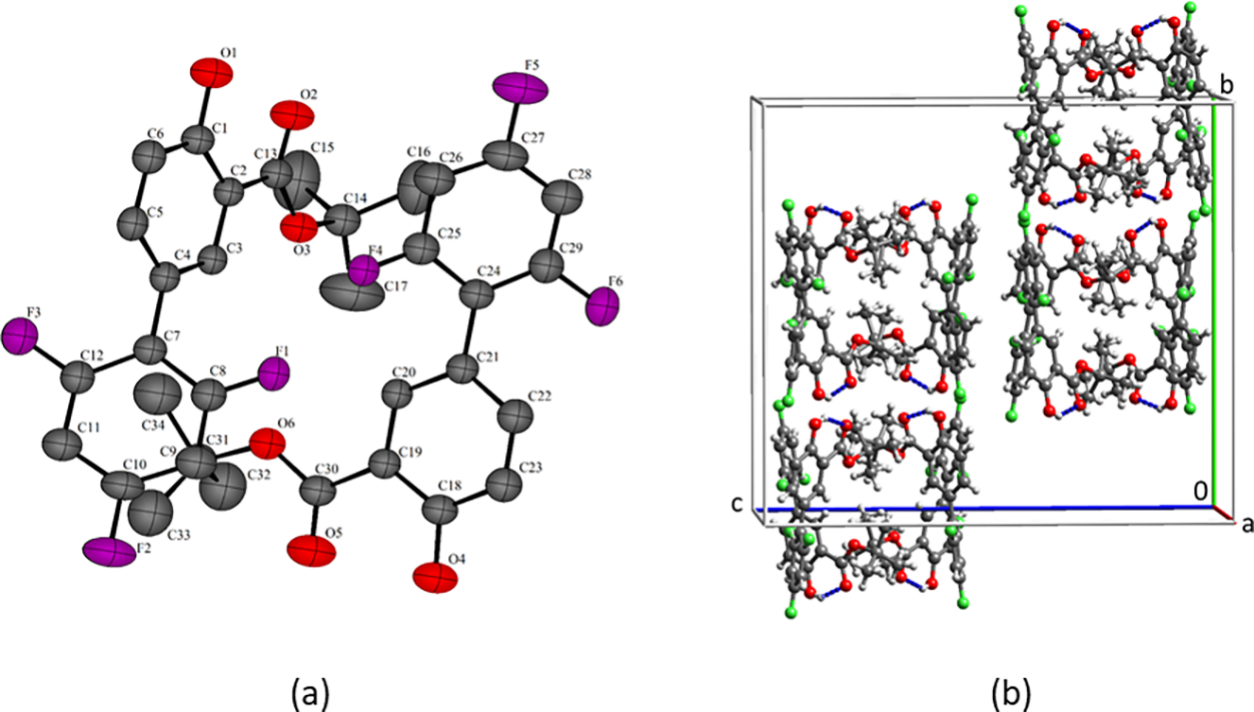
(a) Asymmetric unit of diflunisal t-butyl ester and (b) packed units cell. One component of the ortho-F disorder is shown, and H atoms not in H-bonds have been omitted for clarity.

In complete contrast to diflunisal and the other esters, the tertiary butyl ester does not have a stacked structure and it crystallizes as blocks. The asymmetric unit of the tertiary butyl ester is shown in Figure 5a. The strongest intermolecular interaction in the lattice at −47 kJ/mol is between the molecules in the asymmetric unit and is more than twice that of the next largest interaction. The tertiary butyl groups are too large to allow efficient packing in a stacked structure. In the packing diagram in Figure 5b, a (020) slip plane is clearly present in the structure.

### Persistence of Needle Growth of Diflunisal Methyl and Ethyl Ester

**Figure 6:**
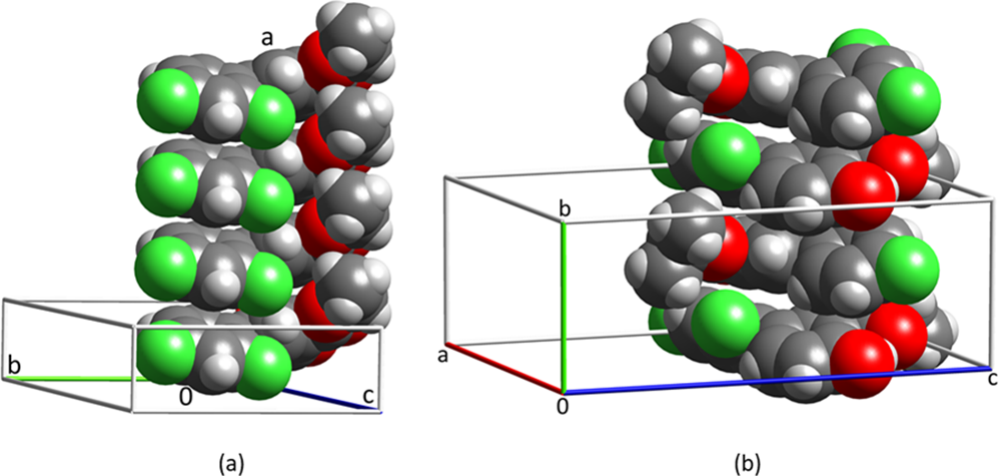
Stacking in the structures of (a) the ethyl ester and (b) the isopropyl ester of diflunisal.

It has been reported that nitromethane has the ability to block the growth of needle crystals in the cases of PABA and lovastatin.^42,43^ It has been suggested that nitromethane has an ability to delaminate stacked structures.^43^ It was found that when diflunisal and its methyl, ethyl, and isopropyl esters were crystallized from nitromethane all grew as needles except the isopropyl ester which grew as blocks. This is observed despite the higher interaction energy within the stacking motif of the latter, Table 2. We attribute this lack of persistent needle growth in the isopropyl case to increased opportunities for solvent interactions provided by the structure with alternating isopropyl groups, Figure 6.

The simple stacking in the case of the ethyl ester is due to the presence of a monolayer in its crystal structure which is normal to the 1D stacking motif, Figure 6a. In structures where the filled unit cell is a monolayer the stacked molecules are symmetry related by a unit translation in the stacking direction.

### Diflunisal Amide Solvate

Diflunisal amide solvate crystallizes as needles from acetonitrile. It has a stacked structure with an intermolecular energy of −30.4 kJ/mol between stack neighbors and more than 80% of the atoms are in vdW contact; more details are in the SI.

### Needle Growth of 4-Hydroxy-*N*-phenylbenzenesulfonamide

**Figure 7:**
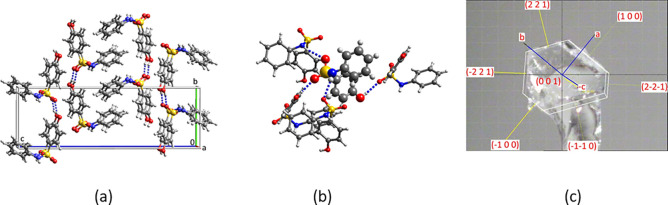
H-bonding in the crystal structures of (a) HPS1, (b) HPS2, and (c) HPS2 crystal indexed on the diffractometer.

**Figure 8:**
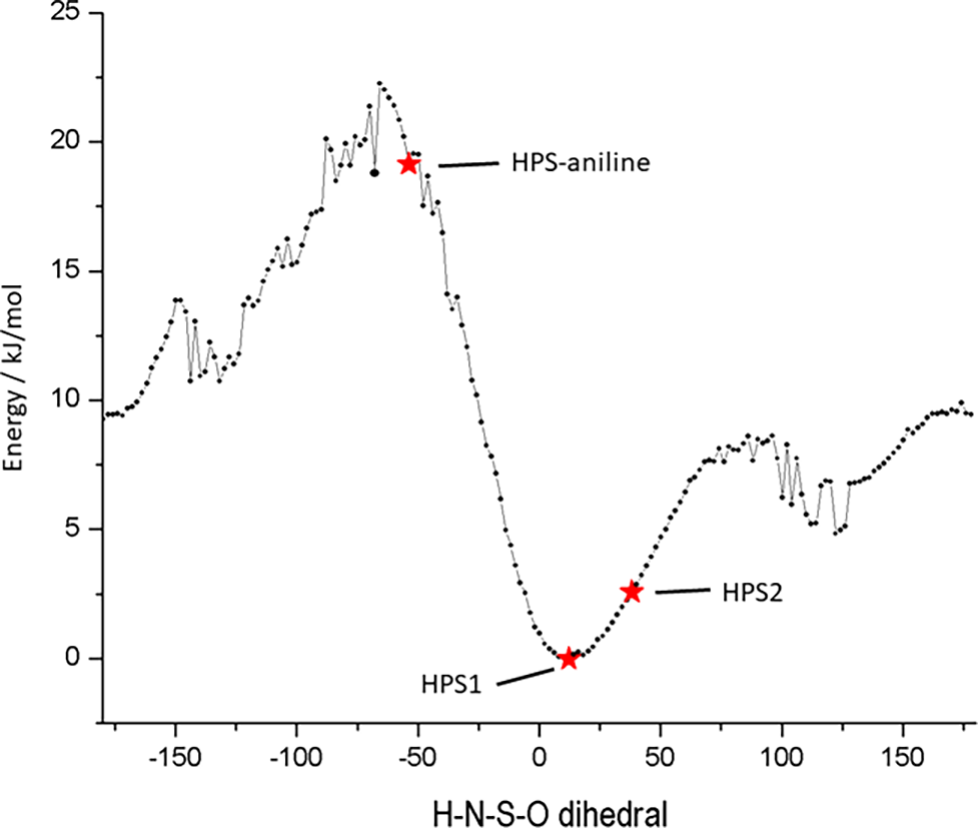
Energy profile plot for rotation about the H–N–S–O dihedral of HPS.

4-Hydroxy-*N*-phenylbenzenesulfonamide (HPS1) has been reported to crystallize as needles both from solution and by sublimation.^44^ We now report that flash cooling of dichloromethane solutions yields a second polymorph, HPS2, which crystallizes as blocks, Figure 7c. Why is the strong tendency toward needle growth observed for HPS1 absent in HPS2? The crystal structure of HPS2 is compared with that of HPS1 in Figure 7. In the HPS1 polymorph, each molecule is H-bonded to two others and the H-bonded chains are stacked in the direction of needle growth along the *a* axis with 52% vdW contact between stacked neighbors.^44^ In the HPS2 structure, each molecule is H-bonded to four others in a 3D arrangement which maximizes H-bonding. An AM1 energy profile plot was calculated for rotation about the H–N–S–O dihedral. The HPS structure was first optimized and then the profile shown in Figure 8 was calculated with all atoms in the structure being optimized except the four atoms defining the dihedral. The angle in the HPS2 structure obtained by flash cooling and that of HPS1 are both close to the minimum energy. The relative energy difference is small so that in this case the rotational angle may not be decisive. On the basis of the density rule^45^ HPS2 may be the kinetic product, and HPS1, the more stable polymorph. Their densities are 1.382 and 1.423 g/cm^3^, respectively. However, it should be noted that a recent systematic analysis suggested that 45% of a set of examples of monotropic phases disobey the density rule.^46^

### 4-Hydroxy-*N*-phenylbenzenesulfonamide Aniline

**Figure 9:**
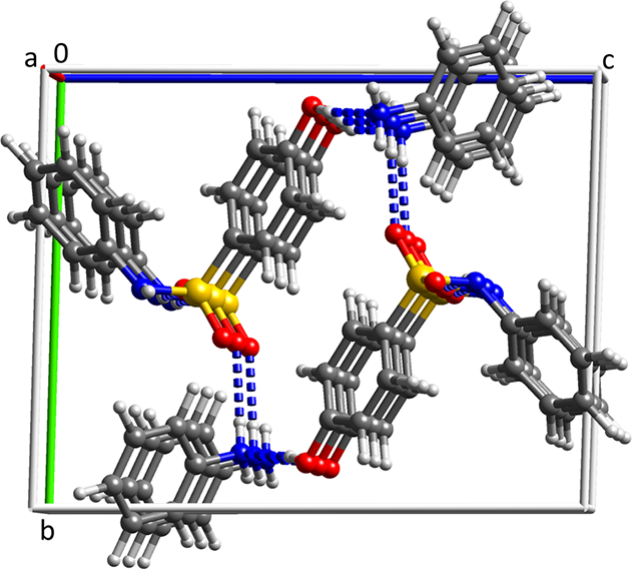
HPS aniline solvate structure viewed down the *a* axis.

The crystal structure of the HPS aniline solvate is shown in Figure 9. The molecules are stacked along the short *a* axis, and the compound crystallized as needles. The mainly dispersive interaction between the HPS molecules in the stacks is greater than in the HPS1 structure, and the HPS molecule adopts a flatter geometry with a flatness index of 0.53 compared to 0.75 for HPS in the HPS1 structure (see the SI for the definition of the flatness index). The H–N–S–O dihedral has a value of −50.3° which takes the molecule close to the highest point of the energy profile plot, Figure 8.

### 2′-Hydroxy[1,1′-bicyclohexyl]-1-carbonitrile

**Figure 10:**
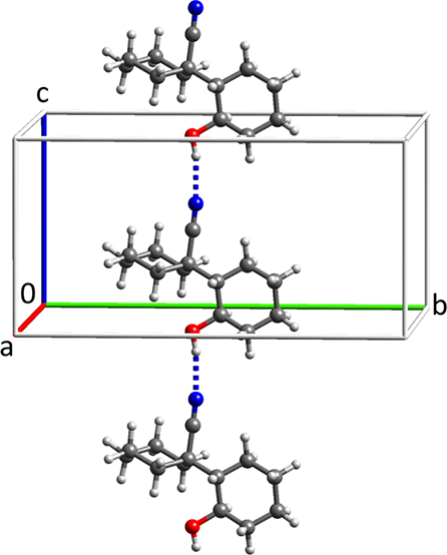
H-bonding in the crystal structure of HBCN.

2′-Hydroxy[1,1′-bicyclohexyl]-1-carbonitrile, HBCN, was found to crystallize as needles from nonhydroxylic solvents like dichloromethane and as blocks from ethanol. Crystal data are in Table S1, and more details are in the SI. The crystal structure contains a 1D H-bond motif which is in the direction of needle growth, Figure 10. The interaction energy between the molecules in the H-bonded chain is −32.1 kJ/mol and just 3% of the atoms are in vdW contact. Hydroxylic solvents are able to suppress needle growth in this case. Hydroxylic solvents can swamp the 1D directional H-bond and thus block the rapid growth in the needle direction.

### Can the Propensity to Yield Needle Crystals Be Quantified?

The crystal structures above which grow as needles from the solvents examined all have stacked structures with a stacking interaction that is greater than −30 kJ/mol, 50% or more of their atoms in vdW contact within the stack, and filled unit cells which are monolayers. We classify these systems as persistent needle formers. Both HBCN and the isopropyl ester of diflunisal (with 3% vdW contact and a double layer unit cell respectively) do not have all of these properties and are therefore classified as controllable needle formers. To see if it is possible to extend this classification of needle forming tendency to a wider range of systems, we have examined literature examples of compounds known to have polymorphs which exhibit needle growth and combined them with the compounds described above in Table 2. It was only possible to use examples from the literature where the needle growth direction was clearly established. This requirement greatly limited the number of examples that could be included. The properties listed in Table 2 are

(i) the presence of a dominant 1D motif in the structure which involves either stacking or H-bonding (or both), and the interaction energy within the motif
(ii) in stacked structures the percent of the atoms in a molecule that are in vdW contact with their stack neighbors
(iii) molecular flatness defined as height/length; a flat molecule has a flatness value close to zero and a spherical molecule will have a value of 1; nonflat molecules which have a high percent vdW contact in stacks are necessarily well fitted into each other;^47^ more details are in the SI
(iv) a packed unit cell forms a monolayer normal to the dominant 1D motif leading to simple stacking
(v) the packing index, an indication of a well packed structure
(vi) 1D motif aligned with the unit cell

### Literature Examples of Compounds That Crystallize As Needles

#### Thymine

**Figure 11:**
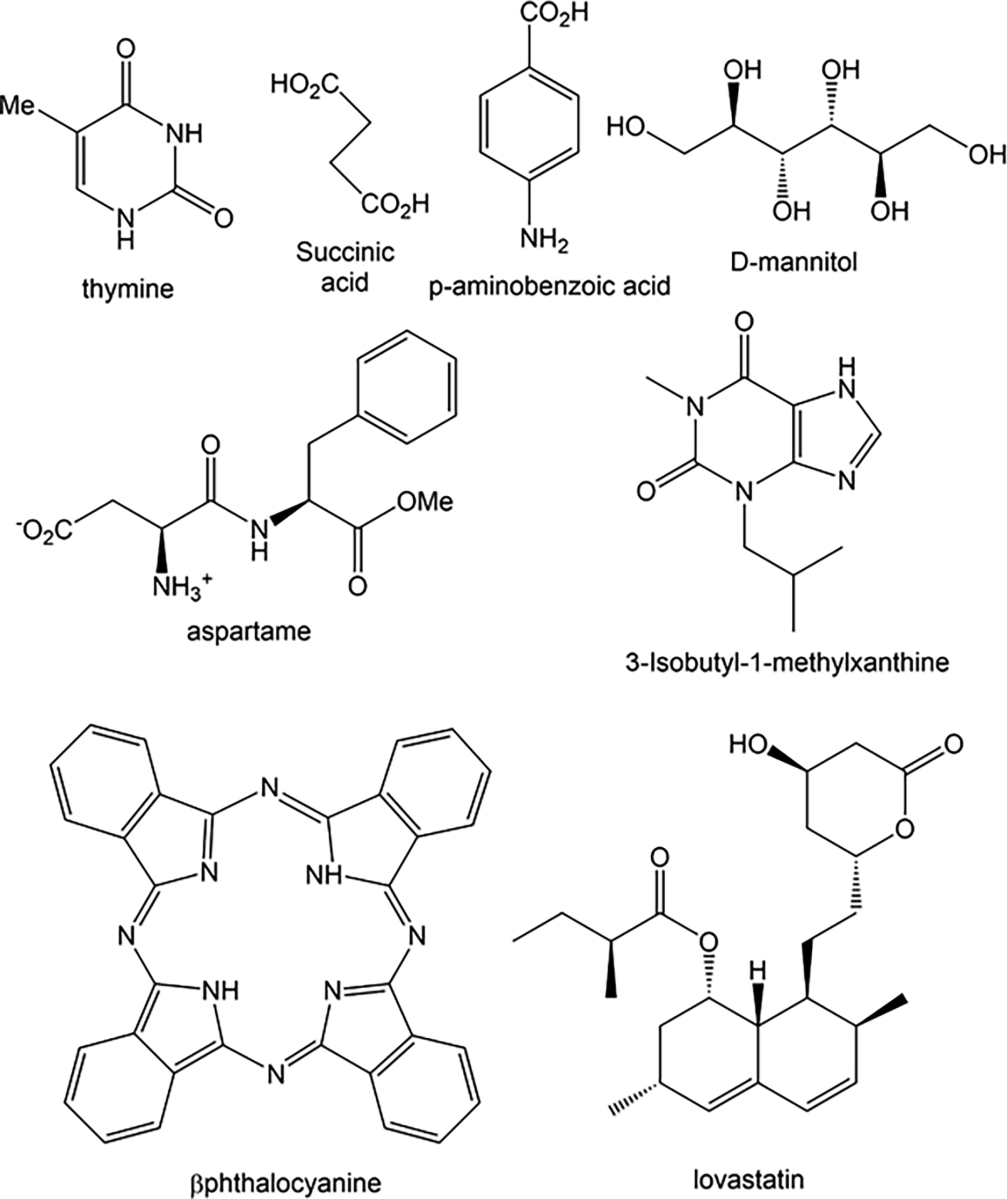
Structural formulas of compounds that give needle crystals.

**Figure 12:**
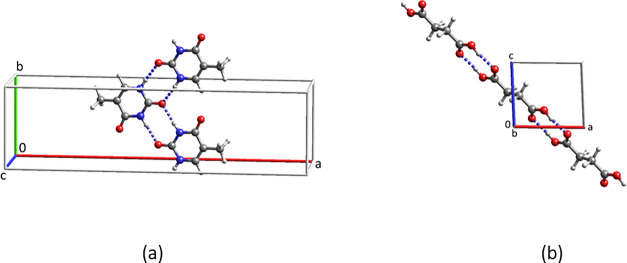
H-bonding in (a) the crystal structure of thymine and (b) succinic acid (view down *b*).

Thymine (Figure 11) crystallizes from 90% H_2_O/ethanol as needles,^60^ as prisms from ethanol, and as plates by sublimation.^61^ The crystal structure contains a doubly bonded H-bond motif along the *b* axis with an interaction energy of −74.8 kJ/mol between the molecules in the chains, Figure 12a. The fraction of atoms in vdW contact within the chains is just 20% which is not sufficient to make needle growth persistent, and crystal growth can be controlled by solvent choice.

#### Succinic Acid

Succinic acid crystals grow by sublimation as blocks, from H_2_O as plates, and as needles from isopropanol.^62^ The H-bonded chains are parallel to the *ac* diagonal, Figure 12b. The 1D motif is not aligned with the unit cell, and needle growth can be controlled by solvent choice.

#### d-Mannitol

**Figure 13:**
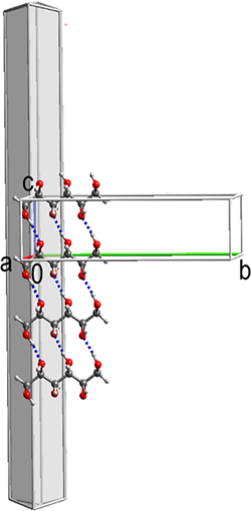
Triple H-bond 1D motif of d-mannitol and crystal growth along *c*.

d-Mannitol grows from H_2_O as needles with extended growth along *c*, Figure 13.^63^ Needle growth is also observed from nitromethane. The triple H-bond with an interaction energy between the molecules of −99.9 kJ/mol combined with 56% vdW contact between the molecules in the 1D motif ensures that needle growth along *c* is persistent. It is also important to note that d-mannitol is not flat. Its flatness index is 0.42, and high vdW contact suggests that the molecules are fitted into each other.

#### Aspartame

**Figure 14:**
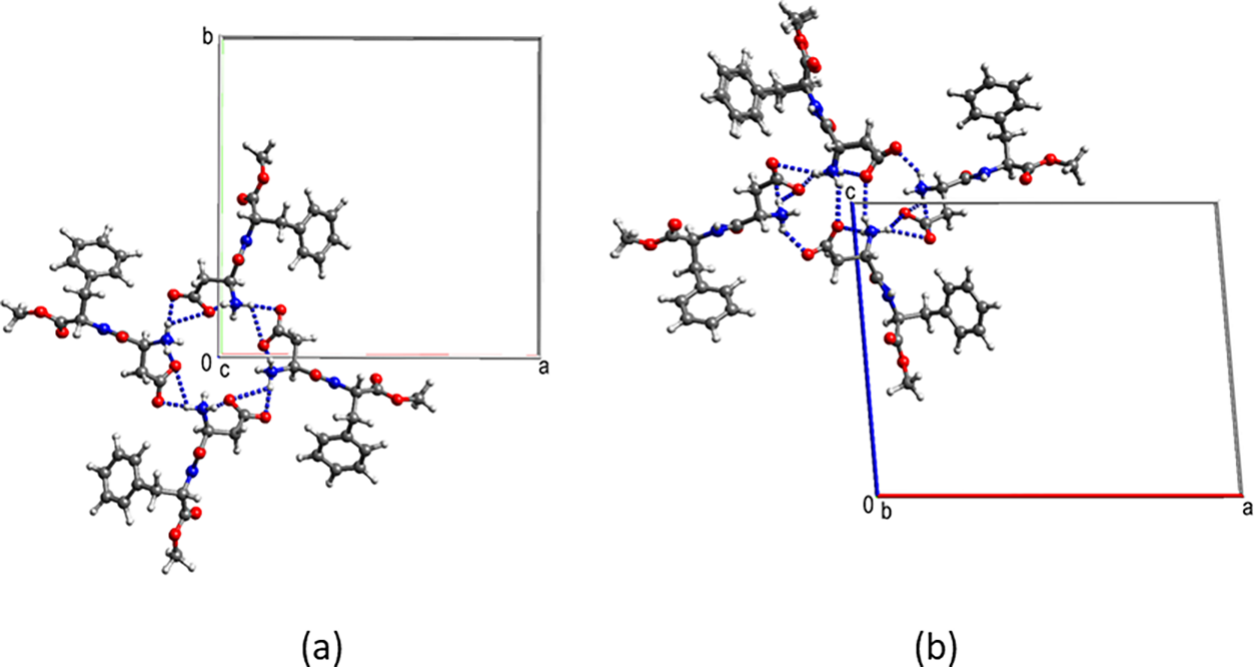
(a) Aspartame hemihydrate, view down the *c* axis of one 4_1_ screw axis, and (b) anhydrous aspartame, view down *b* of one 2_1_ screw axis.

Aspartame hemihydrate has a very strong tendency toward the growth of fine needles, and it was only after considerable effort that needles of sufficient thickness could be obtained for study by X-ray diffraction.^52^ The molecules crystallize in the space group *P*4_1_ with 0.5H_2_O. The zwitterionic molecules are H-bonded in a spiral along the 4_1_ screw axis at each cell corner, Figure 14.

The water molecule is located in a channel down along *c*. It makes a limited contribution to the overall charge assisted H-bonding, and it does not play a crucial role in the structure. The water molecule was therefore removed to make the PIXEL calculations possible. The strong charge assisted H-bonding with an interaction energy of −136.3 kJ/mol combined with 57% of the atoms in vdW contact in the 1D motif strongly favors needle growth along the *c* axis. There are no H-bonds between the stacks.

The crystal structure of the anhydrous form was determined from powder data,^13^ and it is closely related to that of the hemihydrate with similar charge assisted H-bonding supported by vdW contact between the molecules in the 1D motif. Both of these aspartame polymorphs are predicted to be persistent needle formers, and so far, only needle morphology has been reported in the literature.

#### 3-Isobutyl-1-methylxanthine

**Figure 15:**
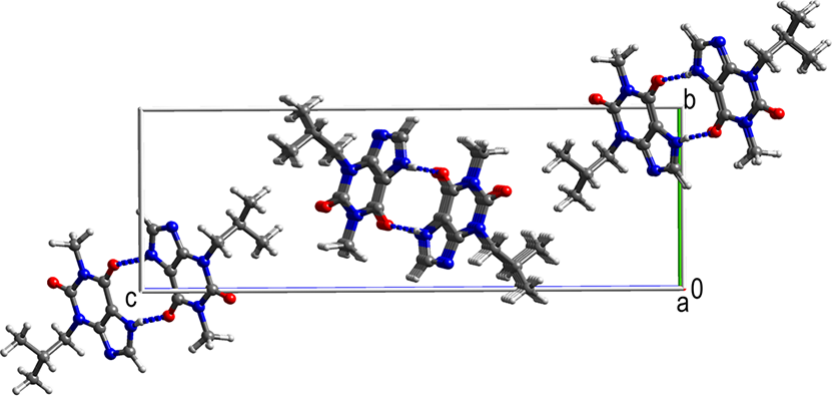
Crystal structure of 3-isobutyl-1-methylxanthine.

3-Isobutyl-1-methylxanthine crystallizes from aqueous methanol as very fine needles.^54^ The crystal structure contains H-bonded dimers which are stacked along the short *a* axis, Figure 15. The interaction energy between the molecules in the stacks is −32 kJ/mol and with a vdW contact fraction of 62% needle growth is predicted to be persistent.

#### *p*-Aminobenzoic Acid

**Figure 16:**
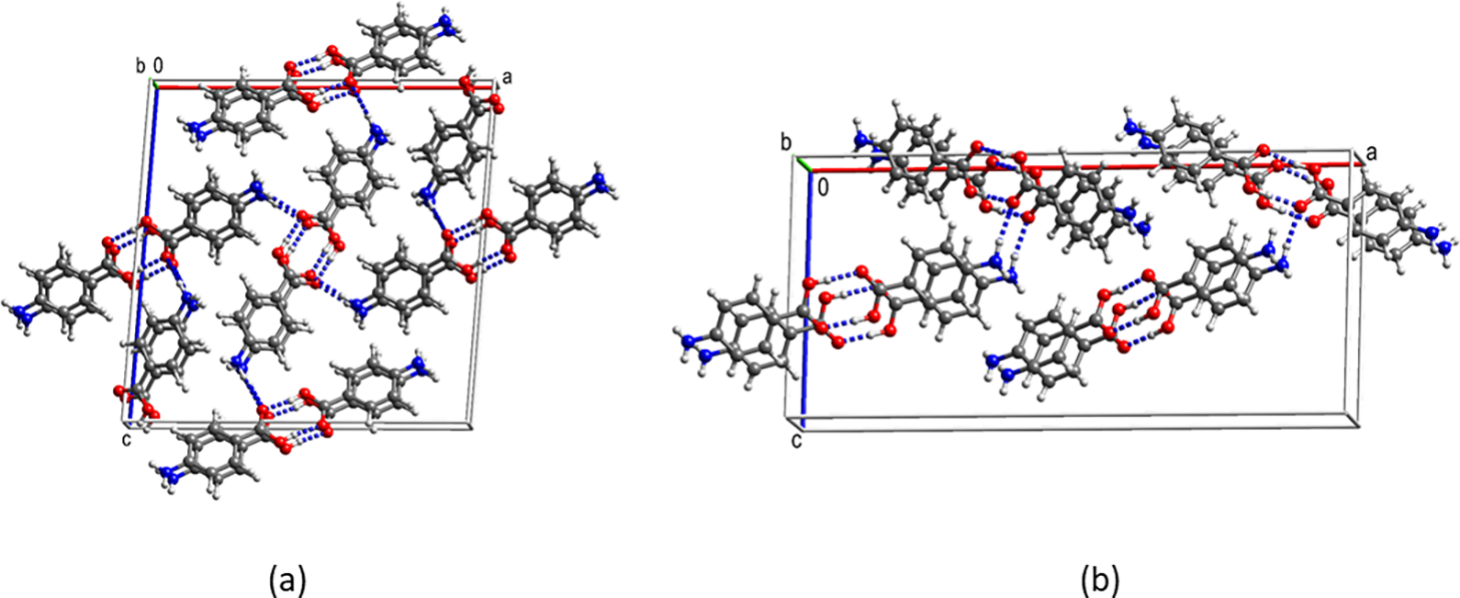
Structure of PABA viewed down *b* (a) form I and (b) form V.

PABA form I has been reported to grow as needles from a range of solvents,^10^ and form V was obtained as needles from an aqueous solution containing selenous acid.^55^ Both of these forms have stacked structures which are stacked in the direction of needle growth, Figure 16. In both cases the fraction of atoms in vdW contact within the stacks is close to 80%; however, the interaction energy between stack neighbors is low at −14.2 and −14.3 kJ/mol, respectively, and these values are just not high enough to ensure persistent needle growth. It has recently been shown that block like crystals are obtained from nitromethane.^42^

#### *m*-Nitroaniline and 4-Nitro-4′-methyl Benzylidene Aniline

**Figure 17:**
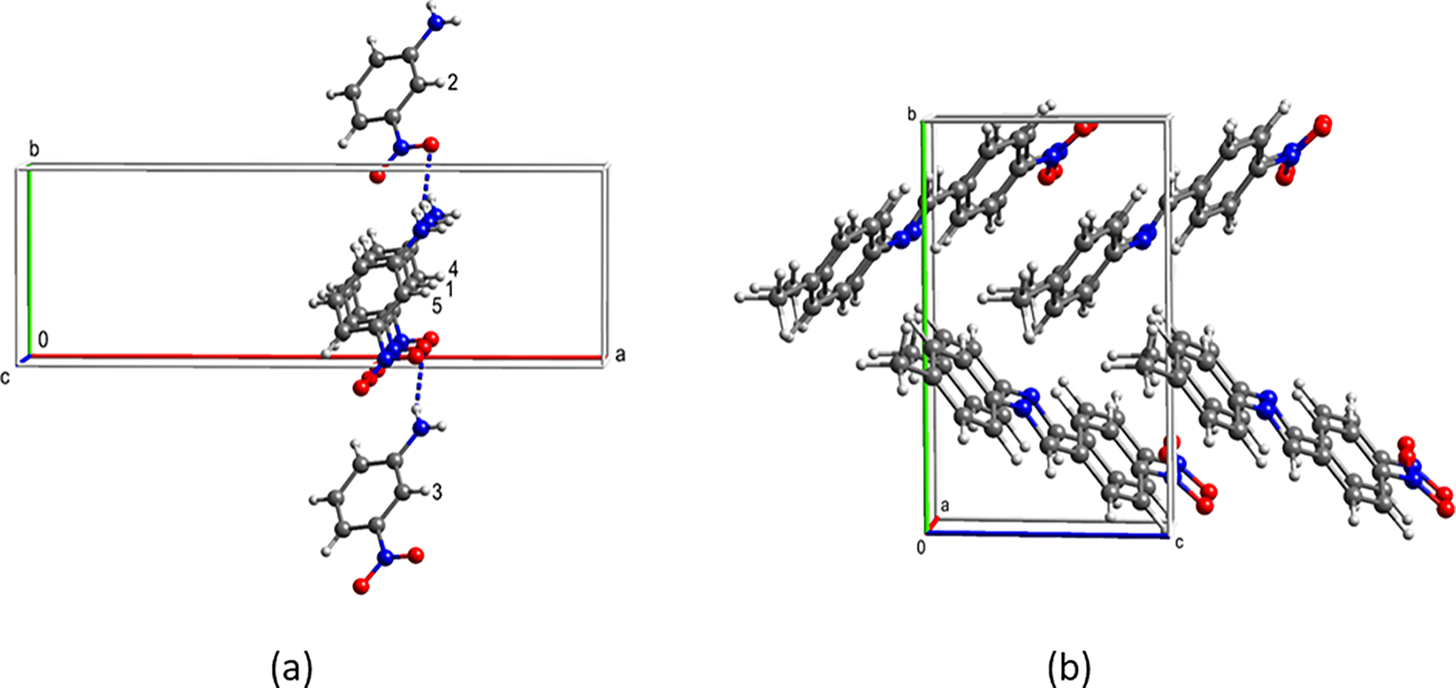
(a) MNA H-bond between molecules 3, 1, and 2 and molecules 5, 1, and 4 are stacked and (b) stacking in NMBA.

*m*-Nitroaniline, MNA, and 4-nitro-4′-methyl benzylidene aniline, NMBA, are nonlinear optical materials which have similar crystal growth patterns. Both show low solubility in *n*-hexane from which they crystallize as needles.^64,65^ The strongest interaction in the *m*-nitroaniline structure, −25.2 kJ/mol, is a 1D H-bond parallel to the *bc* diagonal, Figure 17a, which does not influence crystal growth. It is the weaker stacking interaction along *c* which drives needle growth. From other solvents in which they are more soluble including CCl_4_, methanol, and toluene, the crystals have a more equant shape. The stacking interaction along *a* in NMBA (Figure 17b) has an energy of −21.2 kJ/mol. MNA and NMBA are controllable needle formers.

#### β-Phthalocyanine

**Figure 18:**
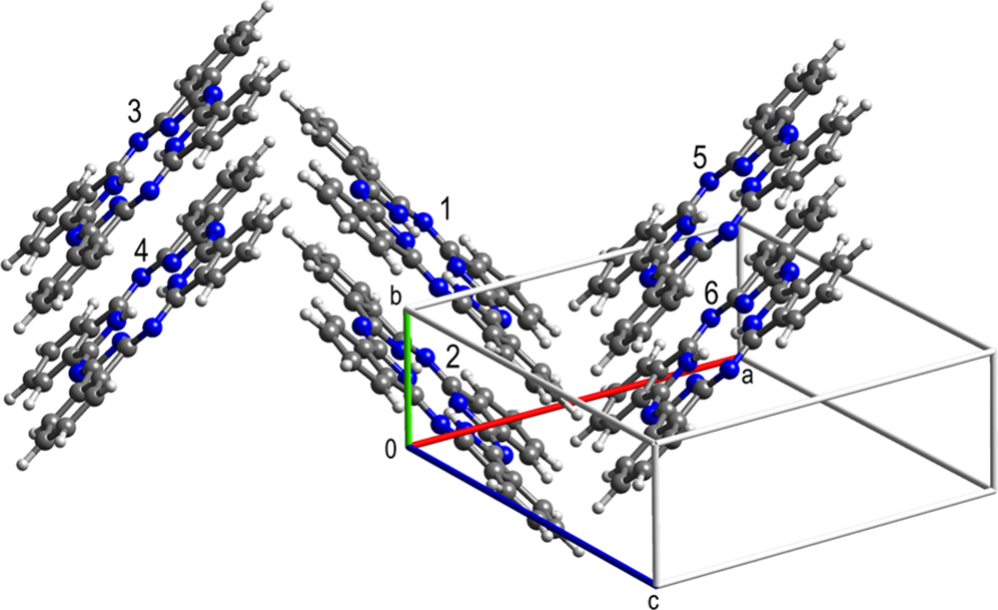
Crystal structure of β-phthalocyanine.

β-Phthalocyanine crystallizes in space group *P*2_1_/*n* with a half molecule in the asymmetric unit. The molecule was completed and the space group was reduced to *P*2_1_ to make PIXEL calculations possible. In the crystal structure the molecules are in slipped stacks. In Figure 18, molecules 1 and 2 are in a stack and molecules 3–6 are the closest contacts to molecule 1 in neighboring stacks. The interaction energies between molecule 1 and molecules 2–6 are −101.5, −29.4, −29.4, −27.6, and −27.6 kJ mol^–1^, respectively. It is the strong dispersion dominated 1D interaction within the stacks with 71% vdW contact between the molecules which drives the persistent needle growth along the short *b* axis; more details are in the SI.

#### Lovastatin

**Figure 19:**
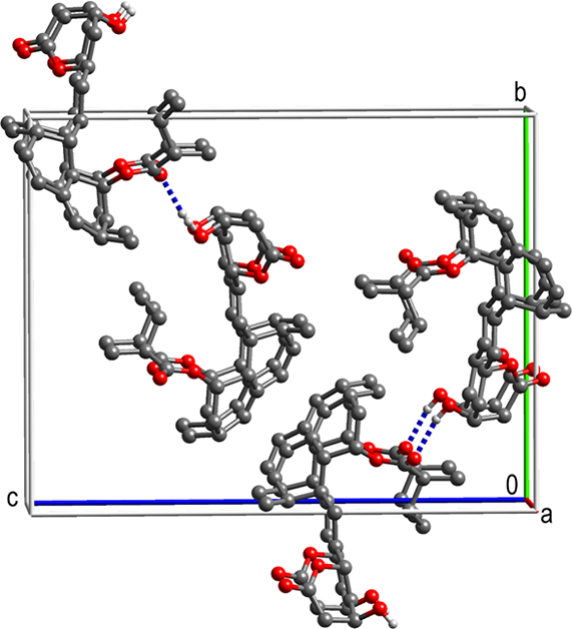
Crystal structure of lovastatin viewed down the stacks.

Lovastatin has been reported to crystallize as needles from alcohols and as rods from ethyl acetate. The crystal structure viewed down the *a* axis shows that there is a 1D stacking motif present, Figure 19. The strongest interaction in the lattice is between the molecules within the stacks. However, the fraction of atoms in vdW contact at 42% is low enough to allow nonhydroxylic solvents especially nitromethane^43^ to delaminate the stacks, control needle formation, and yield a more equant crystal shape.

### Simulation of Crystal Dissolution

Molecular dynamics simulation of pharmaceutical type crystal dissolution has been used to compare different force fields and to estimate heats of solution for aspirin, ibuprofen, and paracetamol.^66^ Amber and Charmm force fields were found to give reasonable results. The mechanism of the dissolution of aspirin crystals has been compared to experimental observations using 4079 molecule clusters.^67^ A molecular dynamics study of the dissolution of *p*-aminobenzoic acid found that clusters of less than 300 molecules were not stable in aqueous solution.^68^ Larger clusters of up to 504 molecules were stable at 0 °C but less stable at 50 and 100 °C.

We were interested to see if simulation of crystal dissolution could reproduce the observation that needle crystals get shorter faster than they get thinner as they dissolve. This should be seen as a faster rate of dissolution of the tops of the molecular stacks in stacked structures than at the stack sides.

Simulations were carried out using Yasara with the Amber14 force field.^69,70^

### Diflunisal Ethyl Ester

**Figure 20:**
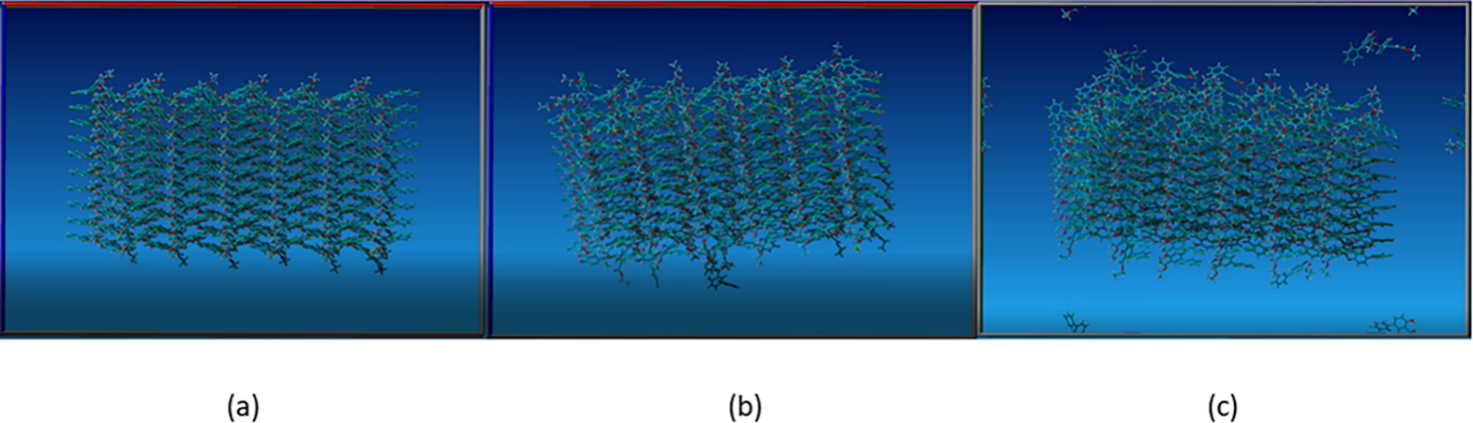
(a) Diflunisal ethyl ester supercell 0 ps, (b) simulation in EtOH after 3000 ps, and (c) simulation in MeNO_2_ after 3000 ps.

A 240 molecule cluster in a simulation box with 4675 molecules of ethanol was used. After 800 ps, molecules leave the both ends of the stacks. After 3000 ps there is considerable disruption of the ends of the stacks. Using the same 240 molecule cluster in a simulation box with 4686 molecules of nitromethane after 300 ps, molecules begin to leave from the ends of the stacks and at 3000 ps a much larger number of molecules have left the cluster than in the ethanol run, Figure 20. There are dissolution movies in the SI. This simulation reproduces the observation that dissolving needles get shorter before they get thinner and the delaminating effect of nitromethane.

### PABA Form I

**Figure 21:**
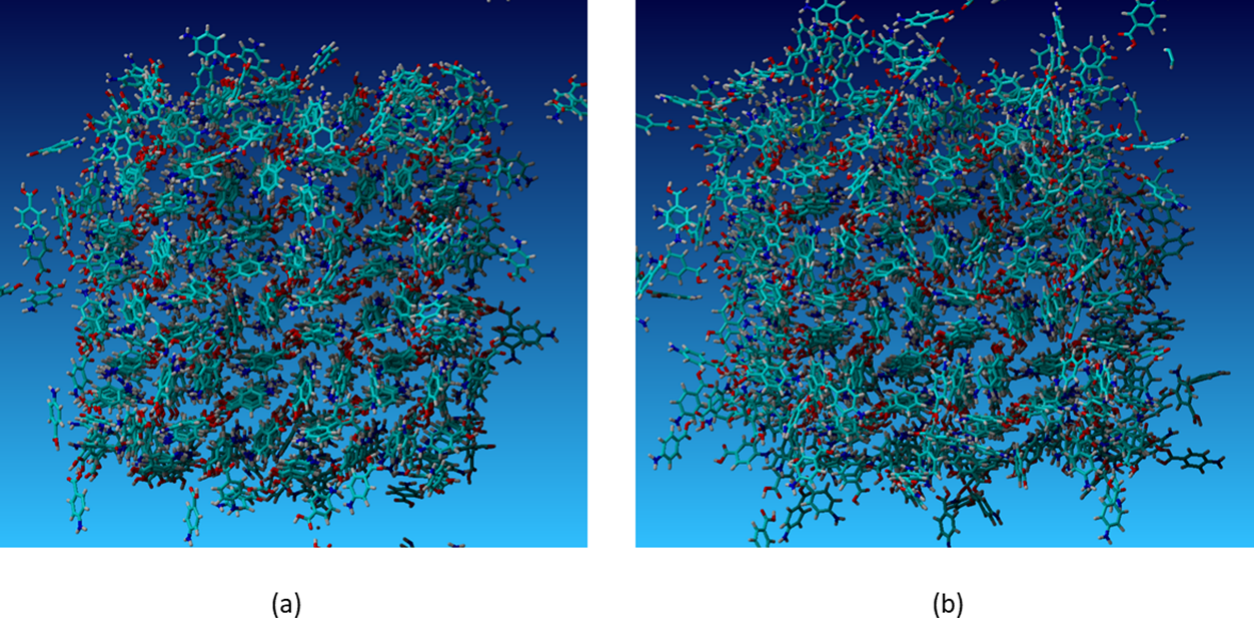
Simulation of PABA form I dissolution after 3000 ps in (a) ethanol and (b) nitromethane.

A 576 molecule supercell of PABA form I in a simulation box with 4288 molecules of ethanol was compared to the same super cell with 4480 molecules of nitromethane. In both cases rapid dissolution took place and after 3000 ps the stacks are more disrupted in nitromethane than in ethanol, Figure 21. This would seem to support the suggestion that nitromethane delaminates π-stacked PABA form I.^42^

### Needle Growth and Needle Dissolution

It has been stressed above that the strength of the bonding within the 1D motif is an important factor driving needle growth. It thus might appear reasonable to argue that these strong intermolecular interactions within the 1D motif would slow dissolution at the top and bottom of the stacks. However, due to the high fraction of atoms in the molecules that are in vdW contact within the stacks the same fraction will be exposed to solvent interactions at the end of the stacks. It is this labilization of the exposed molecules at the stack ends that contributes to making the processes of needle growth and dissolution reversible.

### Classification of Needle Crystals

Needle crystals have been classified as being either absolute or conditional needles. Absolute needles being those that will grow as needles from all solvents tested and conditional needles have aspect ratios which depend on solvent.^6^ It was originally suggested that PABA form I was an example of an absolute needle;^10^ however, subsequent work based on morphology predicted using periodic bond chain analysis combined with smooth growth mechanisms suggested that it was a conditional needle.^6^

We propose the use of the terms persistent and controllable for systems which crystallize with a needle morphology. We have shown that systems classified as persistent needle formers have a consistent set of properties and that systems which do not have these properties have morphologies which can be controlled by solvent choice. The most important properties which drive needle growth are stacking within a 1D motif with more than −30 kJ/mol interaction energy and at least 50% vdW contact and a monolayer filled unit cell. The only effective way to eliminate persistent needle growth is to find another nonstacked polymorph or to introduce a substituent into the structure which hinders molecular stacking.

## Conclusions

The amide and methyl, ethyl, and isopropyl esters of diflunisal crystallize as needles from ethanol. The t-butyl ester crystallizes as blocks from all solvents examined. Of these compounds the t-butyl ester is the only one that does not have a 1D stacking motif in its structure.

Needle growth is reversible in that on dissolution or sublimation needle crystals get shorter faster than they get thinner. This observation has been reproduced by molecular dynamics simulation of dissolution.

An analysis of intermolecular energies calculated using the PIXEL program suggests that the interaction energy within the 1D motif has an important influence on the persistence of needle growth from a range of solvents.

The structures of known needle forming systems from the literature were added to the structures reported here, and the crystal structural features required to drive persistent needle formation were found to be a stacked structure with a stacking interaction that is greater than −30 kJ/mol, 50% or more of their atoms in vdW contact within the stack, and filled unit cells which were monolayers.

Compounds whose structures have some but not all of these properties can crystallize as needles from some solvents and as blocks from others.

This permits the classification of crystal structures into persistent and controllable needle formers. To stop needle growth by persistent needle formers it is necessary to find a nonstacked polymorph or to synthesize a derivative with a substituent which blocks stacking in the crystal structure.

## Supporting Information Available

Experimental details, crystal data, PIXEL data for diflunisal esters, 2′-hydroxy[1,1′-bicyclohexyl]-1-carbonitrile, HPS1, HPS2, and literature compounds which form needles (PDF)
Dissolution simulation of diflunisal ethyl ester in ethanol (MP4)
Dissolution simulation of PABA form I in nitromethane (MP4)

## Accession Codes

CCDC 2064185–2064192 contain the supplementary crystallographic data for this paper. These data can be obtained free of charge via www.ccdc.cam.ac.uk/data_request/cif or by emailing data_request@ccdc.cam.ac.uk, or by contacting The Cambridge Crystallographic Data Centre, 12 Union Road, Cambridge CB2 1EZ, UK; fax: +44 1223 336033.
