# Supplementary material for: Factors Controlling Persistent Needle Crystal Growth: The Importance of Dominant One-Dimensional Secondary Bonding, Stacked Structures, and van der Waals Contact
Source: Cryst Growth Des. 2021 May 21;21(6):3449–60. doi: 10.1021/acs.cgd.1c00217 (PMC8273860; doi:10.1021/acs.cgd.1c00217)
Supplement: Supplementary file 1 — cg1c00217_si_001.pdf [file cg1c00217_si_001.pdf]

Factors Controlling Persistent Needle Crystal Growth: The Importance of Dominant 1D Secondary Bonding, Stacked Structures and vdW Contact

Francesco Civati,[1,2] Ciaran O'Malley,[1] Andrea Erxleben,[1,2,*] and Patrick McArdle[1,*]

[1] School of Chemistry, National University of Ireland, Galway, H91TK33, Ireland

[2] Synthesis and Solid State Pharmaceutical Centre (SSPC), Limerick, V94T9PX, Ireland

*Corresponding author email address: andrea.erxleben@nuigalway.ie (AE); p.mcardle@nuigalway.ie (PM)

Supporting information

Contents

Experimental 1

Crystal data 2

Pixel data for diflunisal esters 5

Pixel data for diflunisal amide solvate 8

Pixel data for 2′-hydroxy[1,1′-bicyclohexyl]-1-carbonitrile, HBCN 8

Pixel data for 4-hydroxy-N-phenylbenzenesulfonamide HPS1 and HPS2 9

Pixel data for literature compounds which form needles 10

References 18

Experimental

Synthesis of diflunisal esters

Synthesis of the diflunisal ester family was carried out following experimental procedures reported in the literature. Diflunisal-methylester, (methyl-2',4'-difluoro-4-hydroxybiphenyl-3-carboxylate) was obtained in 65% yield.[1] Diflunisal-ethylester (ethyl-2',4'-difluoro-4-hydroxybiphenyl-3-carboxylate) was obtained in 82% yield.[2] Diflunisal-isopropylester (isopropyl 2',4'-difluoro-4-hydroxybiphenyl-3-carboxylate) was obtained in 60% yield.[3] Diflunisal-t-butylester (t-butyl 2',4'-difluoro-4-hydroxybiphenyl-3-carboxylate) was obtained in 40 % yield.[4] Diflunisal amide (2',4'-difluoro-4-hydroxy-[1,10- biphenyl]-3-carboxamide) was obtained in 73% yield.[3] Crystallization of diflunisal esters was carried out using slow cooling crystallization from methanol. The diflunisal amide solvate was crystallized from acetonitrile.

4-Hydroxy-*N*-phenylbenzenesulfonamide

4-hydroxy-*N*-phenylbenzenesulfonamide, HPS, was synthesized following a literature procedure.[5] The crystallization of the crude product from dichloromethane led to the formation of a needle-like co-crystal of HPS and aniline. A second recrystallization from dichloromethane led to the formation of needle-like crystals of the thermodynamically stable HPS1, reported as CCDC code VUKRAW.[6] A third recrystallization from dichloromethane using crash cooling with ice water led to the formation of the plate-like crystals of metastable HPS2.

2′-Hydroxy[1,1′-bicyclohexyl]-1-carbonitrile

A sample of 2′-hydroxy[1,1′-bicyclohexyl]-1-carbonitrile, HBCN, was kindly provided by Omolola Gbadebo and Patrick O'Leary.[7,8]

Crystal structure determination and refinement

An Oxford Diffraction Xcalibur system was used to collect X-ray diffraction data at room temperature. The crystal structures were solved using ShelxT and refined using Shelxl within the Oscail package. The Oscail software was also used to obtain the drawings and generate BFDH and slice attachment energy crystal shapes.[9]

Molecular flatness

Molecular flatness is calculated within the Oscail software. It is defined as the molecular height divided by the molecular length. Molecular height is the sum of the distances to the atoms highest above and lowest below the molecular least squares plane. Molecular length is the largest atom-atom distance in the molecule.

Table S1. Crystal data								
Identification code	dif_me	dif_et	dif_ipr	dif_tbu	dif_amide	hps_an	hps2	HBCN
Empirical formula	$C_{14}H_{10}F_2O_3$	$C_{15}H_{12}F_2O_3$	$C_{16}H_{14}F_2O_3$	$C_{17}H_{16}F_2O_3$	$C_{15}H_{12}F_2N_2O_2$	$C_{18}H_{18}N_2O_3S$	$C_{12}H_{11}NO_3S$	$C_{13}H_{21}NO$
Formula weight	264.22	278.25	292.27	306.30	290.27	342.40	249.28	207.31
Temperature	299.8(7) K	300.0(1) K	299.0(1) K	298.1(3) K	297.9(6) K	296.8(9) K	296.0(1) K	299.6(7) K
Wavelength	0.71073 Å	0.71073 Å	0.71073 Å	0.71073 Å	0.71073 Å	0.71073 Å	0.71073 Å	0.71073 Å
Crystal system	Monoclinic	Orthorhombic	Monoclinic	Orthorhombic	Monoclinic	Triclinic	Orthorhombic	Monoclinic
Space group	$P2_1/n$	$P2_12_12_1$	$P2_1/n$	Pbca	$P2_1$	P-1	Pbca	$P2_1/c$
Unit cell dimensions	a = 3.8439(3) Å	a = 3.9020(4) Å	a = 10.4589(16) Å	a = 11.8996(4) Å	a = 10.208(3) Å	a = 5.1614(6) Å	a = 12.2913(4) Å	a = 8.662(2) Å
	b = 11.7497(12) Å	b = 11.8052(14) Å	b = 7.3958(12) Å	b = 21.3610(8) Å	b = 3.8710(9) Å	b = 11.4039(11) Å	b = 9.2086(3) Å	b = 16.684(3) Å
	c = 26.518(2) Å	c = 28.462(3) Å	c = 18.755(3) Å	c = 23.8375(11) Å	c = 17.643(4) Å	c = 14.3537(12) Å	c = 21.1726(7) Å	c = 8.3595(11) Å
	α= 90°.	α= 90°.	α= 90°.	α= 90°.	α= 90°.	α= 91.433(7)°.	α= 90°.	α= 90°.
	β= 91.877(5)°.	β= 90°.	β= 105.330(15)°.	β= 90°.	β= 103.20(3)°.	β= 97.525(8)°.	β= 90°.	β= 95.187(14)°.
	γ = 90°.	γ = 90°.	γ = 90°.	γ = 90°.	γ = 90°.	γ = 94.548(9)°.	γ = 90°.	γ = 90°.
Volume	1197.03(18) $Å^3$	1311.1(3) $Å^3$	1399.1(4) $Å^3$	6059.2(4) $Å^3$	678.8(3) $Å^3$	834.41(15) $Å^3$	2396.44(14) $Å^3$	1203.1(4) $Å^3$
Z	4	4	4	16	2	2	8	4
Density (calculated)	1.466 Mg/m^3	1.410 Mg/m^3	1.388 Mg/m^3	1.343 Mg/m^3	1.420 Mg/m^3	1.363 Mg/m^3	1.382 Mg/m^3	1.145 Mg/m^3
Absorption coefficient	0.123 mm^{-1}	0.116 mm^{-1}	0.112 mm^{-1}	0.107 mm^{-1}	0.114 mm^{-1}	0.213 mm^{-1}	0.265 mm^{-1}	0.071 mm^{-1}

F(000)	544	576	608	2560	300	360	1040	456
Crystal size	0.50 x 0.10 x 0.10 mm^3	0.50 x 0.10 x 0.10 mm^3	0.50 x 0.20 x 0.10 mm^3	0.50 x 0.40 x 0.20 mm^3	0.50 x 0.40 x 0.20 mm^3	0.50 x 0.20 x 0.10 mm^3	0.50 x 0.40 x 0.30 mm^3	0.50 x 0.25 x 0.15 mm^3
Theta range for data collection	3.794 to 29.169°.	3.452 to 29.254°.	3.559 to 29.279°.	3.555 to 29.203°.	3.471 to 29.036°.	3.587 to 29.203°.	3.834 to 29.304°.	3.457 to 29.123°.
Index ranges	-5<=h<=4, - 14<=k<=7, -	-2<=h<=5, - 15<=k<=10, -	-13<=h<=11, - 10<=k<=9, -	-9<=h<=16, - 28<=k<=25, -	-12<=h<=13, - 4<=k<=4, -	-6<=h<=7, - 9<=k<=15, -	-16<=h<=16, - 12<=k<=12, -	-11<=h<=11, - 22<=k<=22, -
Reflections collected	4973	4052	10341	17630	2950	6204	17545	4633
Independent reflections	2759 [R(int) = 0.0270]	2839 [R(int) = 0.0245]	3347 [R(int) = 0.0268]	7154 [R(int) = 0.0226]	2523 [R(int) = 0.0288]	3816 [R(int) = 0.0250]	3039 [R(int) = 0.0276]	4633 [R(int) = 0.0676]
Completeness to theta = 25.242°	99.6 %	99.6 %	99.7 %	99.7 %	99.5 %	99.8 %	99.8 %	99.8 %
Absorption correction	Semi-empirical from equivalents	Semi-empirical from equivalents	Semi-empirical from equivalents	Semi-empirical from equivalents	Semi-empirical from	Semi-empirical from equivalents	Semi-empirical from equivalents	Semi-empirical from equivalents
Max. and min. transmission	1.00000 and 0.64639	1.00000 and 0.84613	1.00000 and 0.71282	1.00000 and 0.75342	1.00000 and 0.08564	1.00000 and 0.92348	1.00000 and 0.93198	1.00000 and 0.85004
Refinement method	Full-matrix least-squares on F^2	Full-matrix least-squares on F^2	Full-matrix least-squares on F^2	Full-matrix least-squares on F^2	Full-matrix least-squares on F^2	Full-matrix least-squares on F^2	Full-matrix least-squares on F^2	Full-matrix least-squares on F^2
Data / restraints / parameters	2759 / 144 / 174	2839 / 0 / 183	3347 / 0 / 193	7154 / 0 / 423	2523 / 157 / 201	3816 / 0 / 230	3039 / 0 / 159	4633 / 121 / 139
Goodness-of-fit on F^2	1.017	1.110	1.031	1.014	1.057	1.007	1.064	0.983
Final R indices [I>2sigma(I)]	R_1 = 0.0492, wR_2 = 0.1112	R_1 = 0.0578, wR_2 = 0.1256	R_1 = 0.0690, wR_2 = 0.1901	R_1 = 0.0556, wR_2 = 0.1335	R_1 = 0.0805, wR_2 = 0.1923	R_1 = 0.0544, wR_2 = 0.1018	R_1 = 0.0431, wR_2 = 0.0970	R_1 = 0.0695, wR_2 = 0.1919
R indices (all data)	R_1 = 0.0774, wR_2 = 0.1287	R_1 = 0.0807, wR_2 = 0.1479	R_1 = 0.1114, wR2 = 0.2281	R_1 = 0.0915, wR_2 = 0.1588	R_1 = 0.1339, wR_2 = 0.2407	R_1 = 0.0968, wR_2 = 0.1194	R_1 = 0.0644, wR_2 = 0.1081	R1 = 0.1307, wR_2 = 0.2146
Largest diff. peak and hole	0.166 and -0.174 e.Å^{-3}	0.196 and -0.184 e.Å^{-3}	0.897 and -0.297 e.Å^{-3}	0.216 and -0.208 e.Å^{-3}	0.233 and -0.262 e.Å^{-3}	0.208 and -0.319 e.Å^{-3}	0.253 and -0.369 e.Å^{-3}	0.508 and -0.272 e.Å^{-3}

Diflunisal methyl ester

The crystal structure of diflunisal methyl ester and a view down the *a* axis are shown in Figure S1 and intermolecular energies are in Table S2. The strongest interaction in the lattice of -33.6 kJ/mol is between stack neighbours along the *a* axis and it is dispersion dominated.

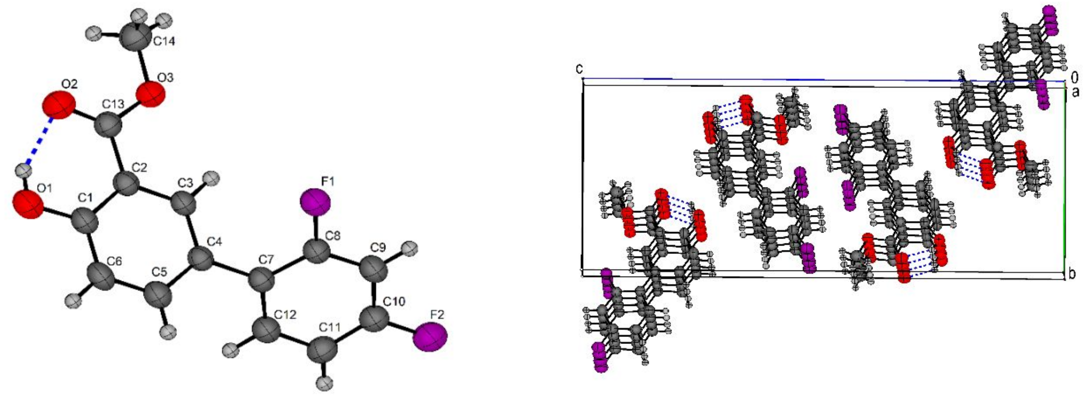

Figure S1. (a) Crystal structure of diflunisal methyl ester, (b) view down *a* axis

Table S2. Intermolecular energies (kJ mol^{-1}) calculated for diflunisal methyl ester using PIXEL

dist.	Coul.	pol.	disp.	rep.	Pixel	Symm Ops	
3.844	-3.5	-4.2	-57.4	31.4	-33.6	1+x,+y,+z	Stack along *a*
3.844	-3.5	-4.2	-57.4	31.4	-33.6	-1+x,+y,+z	Stack along *a*
6.785	-5.1	-1.7	-20.9	6.3	-21.3	1-x,1-y,1-z	Inter stack
8.958	-9.4	-3.2	-13.8	10.8	-15.6	3/2-x,1/2+y,3/2-z	Inter stack
8.958	-9.4	-3.2	-13.8	10.8	-15.6	3/2-x,-1/2+y,3/2-z	Inter stack

Diflunisal ethyl ester

The crystal structure of diflunisal ethyl ester is shown in Figure S2 and intermolecular energies are in Table S3. The strongest interaction in the lattice of -40.5 kJ/mol is between stack neighbours along the *a* axis and it is dispersion dominated.

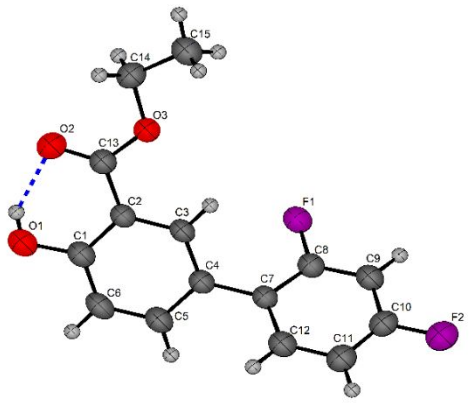

Figure S2. Crystal structure of diflunisal ethyl ester.

Table S3. Intermolecular energies (kJ mol^{-1}) calculated for diflunisal ethyl ester using PIXEL							
dist.	Coul.	pol.	disp.	rep.	Pixel	Symm Ops	
3.902	-6.5	-5.4	-63.6	35.1	-40.5	1+x,+y,+z	Stack along *a*
3.902	-6.5	-5.4	-63.6	35.1	-40.5	-1+x,+y,+z	Stack along *a*
9.145	-9.6	-3.4	-13.8	11.8	-15.1	1-x,1/2+y,1/2-z	Inter stack
9.145	-9.6	-3.4	-13.8	11.8	-15.1	1-x,-1/2+y,1/2-z	Inter stack

Diflunisal i-propyl ester

The crystal structure of diflunisal i-propylester is shown in Figure S3 and intermolecular energies are in Table S4. The strongest interaction in the lattice of -40.5 kJ/mol is between stack neighbours along the *b* axis and it is dispersion dominated. The molecules pack with i-Pr on alternate sides of the stacks to minimize steric problems.

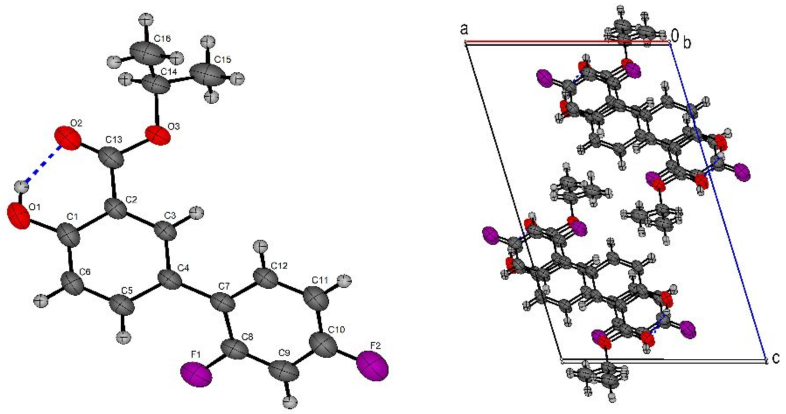

Figure S3. (a) Crystal structure of diflunisal isopropylester and (b) view down the *b* axis.

Table S4. Intermolecular energies (kJ mol^{-1}) calculated for diflunisal isopropylester using PIXEL							
dist.	Coul.	pol.	disp.	rep.	Pixel	Symm Ops	
3.86	-13.1	-5.7	-66	39.4	-45.5	3/2-x,1/2+y,3/2-z	Stack along *b*
3.86	-13.1	-5.7	-66	39.4	-45.5	3/2-x,-1/2+y,3/2-z	Stack along *b*
8.38	-0.5	-2.9	-30	13	-20.5	1-x,1-y,1-z	Inter stack

Diflunisal t-butylester

The crystal structure of diflunisal t-butylester is shown in Figure S4 and intermolecular energies are in Table S5. The strongest interaction in the lattice of only -18.8 kJ/mol is between the pair of molecules in the asymmetric unit.

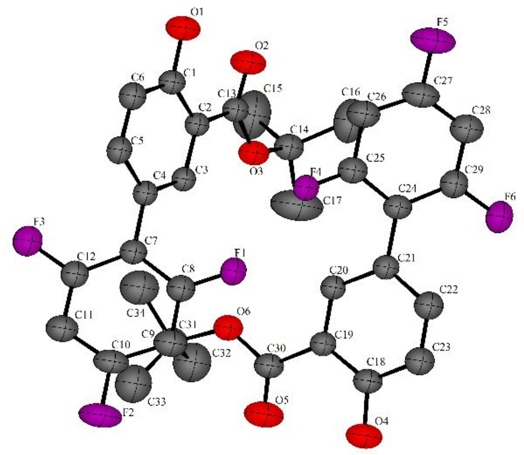

Figure S4. Crystal structure of diflunisal t-butylester with H atoms omitted for clarity

Table S5. Intermolecular energies (kJ mol^{-1}) calculated for diflunisal t-butylester using PIXEL							
dist.	Coul.	pol.	disp.	rep.	Pixel	Symm Ops	
8.37	-5.7	-3.2	-24.6	14.8	-18.8		Intra asymmetric unit contact
6.049	-2.9	-1.3	-20.4	7.5	-17.1	1-x,1-y,1-z	
12.013	-3.5	-1.1	-5.5	2.3	-7.8	1/2-x,1/2+y,+z	

Diflunisal amide solvate

The crystal structure of diflunisal amide solvate is shown in Figure S5 and the intermolecular energies are in Table S6.

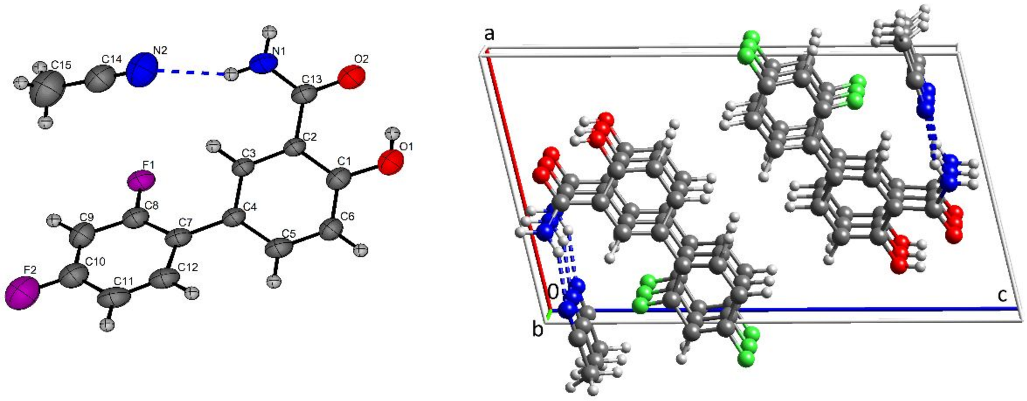

Figure S5. (a) Crystal structure of diflunisal amide solvate and (b) stacking down the *b* axis

The strongest interaction involves the H-bond to the acetonitrile. The 3.871 Å interactions are the dispersion dominated stacking interactions shown in Figure S5(b) and the 11.042 Å interactions involve inter stack interactions.

**Table S6.** Intermolecular energies (kJ mol^{-1}) calculated for diflunisal amide using PIXEL							
dist.	Coul.	pol.	disp.	rep.	Pixel	symm ops	
4.435	-33.4	-12.1	-18.8	27.6	-36.8		As in Figure S5(a)
3.871	-2.4	-3.6	-51.5	27.5	-30.1	x,1+y,+z	Stack along *b*
3.871	-2.4	-3.6	-51.5	27.5	-30.1	x,-1+y,+z	Stack along *b*
11.042	-26.3	-8.3	-10.7	16.4	-28.9	1-x,1/2+y,-z	H-bond to CH_3CN
11.042	-26.3	-8.3	-10.7	16.4	-28.9	1-x,-1/2+y,-z	H-bond to CH_3CN

2′-Hydroxy[1,1′-bicyclohexyl]-1-carbonitrile

The crystal structure of 2′-hydroxy[1,1′-bicyclohexyl]-1-carbonitrile, HBCN, is shown in Figure S6 and intermolecular energies are in Table S7. The strongest interaction in the lattice of -32.4 kJ/mol is between the molecules in the 1D H bonding and it is mostly a Coulombic interaction.

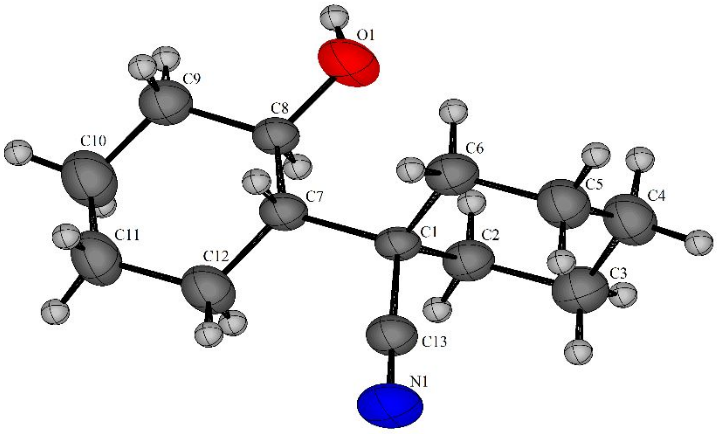

Figure S6. Crystal structure of 2′-hydroxy[1,1′-bicyclohexyl]-1-carbonitrile

**Table S7.** Intermolecular energies (kJ mol^{-1}) calculated using PIXEL							
dist.	Coul.	pol.	disp.	rep.	Pixel	Symm Op	
8.359	-36.8	-14.5	-10.7	29.6	-32.4	+x,+y,1+z	1D H-bond along *c*
8.359	-36.8	-14.5	-10.7	29.6	-32.4	+x,+y,-1+z	1D H-bond along *c*
5.899	-9.1	-3.7	-23.3	7.8	-28.2	2-x,1-y,1-z	33% vdW contact
6.117	-9.7	-3.6	-22.2	10.6	-24.9	1-x,1-y,1-z	33% vdW contact
5.933	-5.3	-4	-34.6	20.6	-23.3	1-x,1-y,-z	36% vdW contact

4-hydroxy-*N*-phenylbenzenesulfonamide HPS1 and HPS2

Pixel data for HPS1 and HPS2 are in Tables S8 and S9.

Table S8 Intermolecular energies (kJ mol^{-1}) calculated for HPS1 using PIXEL

dist.	Coul.	pol.	disp.	rep.	Pixel	Symm Op	
8.631	-36.4	-15	-15.5	26.8	-40.1	+x,1+y,+z	H bond along *b*
8.631	-36.4	-15	-15.5	26.8	-40.1	+x,-1+y,+z	H bond along *b*
5.229	-11.2	-6.2	-38.4	18.8	-37	1+x,+y,+z	Stack along *a*
5.229	-11.2	-6.2	-38.4	18.8	-37	-1+x,+y,+z	Stack along *a*
7.163	-4.1	-3.7	-31.9	15.5	-24.3	1/2+x,1/2-y,-z	Inter stack contact
7.163	-4.1	-3.7	-31.9	15.5	-24.3	-1/2+x,1/2-y,-z	Inter stack contact
8.574	-7.9	-3.8	-22.2	13.7	-20.2	1/2+x,3/2-y,-z	Inter stack contact
8.574	-7.9	-3.8	-22.2	13.7	-20.2	-1/2+x,3/2-y,-z	Inter stack contact

Table S9 Intermolecular energies (kJ mol^{-1}) calculated for HPS2 using PIXEL

dist.	Coul.	pol.	disp.	rep.	Pixel	Symm Op	
7.344	-39.3	-15.8	-28.8	33.8	-50.2	+x,-1+y,+z	H bond
7.344	-39.3	-15.8	-28.8	33.9	-50.1	1+x,+y,+z	H bond
6.035	-29.8	-13.3	-36.3	29.8	-49.5	-1+x,+y,+z	H bond
6.035	-29.8	-13.3	-36.3	29.9	-49.4	1/2+x,1/2-y,-z	H bond
6.512	-8.3	-4	-25.1	9.1	-28.2	-1/2+x,1/2-y,-z	
6.512	-8.3	-4	-25.1	9.1	-28.2	1/2+x,3/2-y,-z	
7.913	-8	-2.2	-22.1	10.7	-21.7	-1/2+x,3/2-y,-z	

Thymine CSD THYMIN03

The crystal structure of thymine is shown in figure S7 and intermolecular energies are in Table S10.

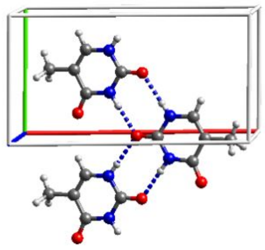

Figure S7. 1D double H-bonded chain parallel to the *b* axis.

**Table S10.** Intermolecular energies (kJ mol^{-1}) calculated for THYMIN03 using PIXEL							
dist.	Coul.	pol.	disp.	rep.	Pixel		
6.636	-84.4	-28.2	-20.7	58.4	-74.8	1-x,2-y,1-z	H-bond chain *b*
5.999	-63	-22.7	-19.4	56.3	-48.9	1-x,1-y,1-z	H-bond chain *b*
4.353	-11.6	-3.8	-18.6	13.1	-20.9	+x,1-y,1/2+z	Adjacent H-bond chain
4.353	-11.6	-3.8	-18.6	13.1	-20.9	+x,1-y,-1/2+z	Adjacent H-bond chain
6.838	-11.6	-3.7	-6.4	6.6	-15.1	+x,1+y,+z	H-bond chain *b*
6.839	-11.6	-3.7	-6.4	6.6	-15.1	+x,-1+y,+z	H-bond chain *b*

Succinic acid CSD SUCCAB18

The crystal structure of succinic acid is shown in Figure S8 and intermolecular energies are in Table S11. The dominant 1D contains strong H-bonds which run along the *ac* diagonal and thus the 1D motif is not aligned with the unit cell.

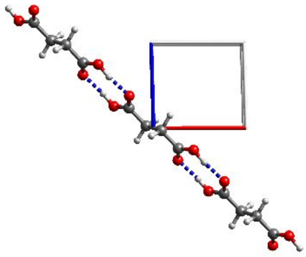

Figure S8. H-bond chain in the succinic acid structure, view down the *b* axis.

**Table S11.** Intermolecular energies (kJ mol-1) calculated for SUCCAB18 using PIXEL							
dist.	Coul.	pol.	disp.	rep.	Pixel	Symm Op	
7.619	-124.4	-59.3	-20.6	129.1	-75.2	1+x,+y,-1+z	H-bond chain
7.619	-124.4	-59.3	-20.6	129.1	-75.2	-1+x,+y,1+z	H-bond chain
5.101	-7.7	-2.4	-12.1	5.7	-16.5	+x,+y,1+z	Inter chain
5.101	-7.7	-2.4	-12.1	5.7	-16.5	+x,+y,-1+z	Inter chain
5.113	-7.6	-2.3	-11.6	8.5	-13.2	+x,3/2-y,1/2+z	Inter chain
5.113	-7.6	-2.3	-11.6	8.5	-13.2	+x,3/2-y,-1/2+z	Inter chain

Aspartame CSD DAWGOX

The crystal structure of aspartame hydrate is shown in Figure S9 and intermolecular energies are in Table S12. The strong interactions, 9.936 and 4.919 Å, are all related by a 4_1 screw axis and are within a stack and the weaker 7.092 Å interaction is between stacks.

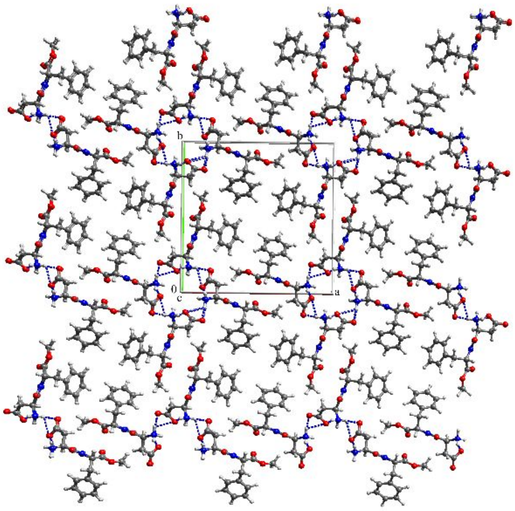

Figure S9. Crystal structure of aspartame hydrate with the water removed for PIXEL calculations

**Table S12.** Intermolecular energies (kJ mol-1) calculated for DAWGOX using PIXEL							
dist.	Coul.	pol.	disp.	rep.	Pixel	Symm Op	
9.936	-124.2	-40.1	-18.9	45.6	-137.6	-y,+x,-3/4+z	H-bond in stack along *c*
9.936	-124.2	-40.1	-18.9	45.6	-137.6	+y,-x,3/4+z	H-bond in stack along *c*
4.919	-92.2	-39.2	-59.5	71.6	-119.3	+x,+y,1+z	H-bond in stack along *c*
4.919	-92.2	-39.2	-59.5	71.6	-119.3	+x,+y,-1+z	H-bond in stack along *c*
7.092	-28	-9.6	-27.7	20.2	-45.1	1-x,-y,1/2+z	Inter stack contact
7.092	-28	-9.6	-27.7	20.2	-45.1	1-x,-y,-1/2+z	Inter stack contact

Aspartame CSD KETXIR

The crystal structure of aspartame is shown in Figure S10 and intermolecular energies are in Table S13. There are two molecules in the asymmetric unit. All of the interactions in the table are within stacks. All inter stack interactions are much weaker.

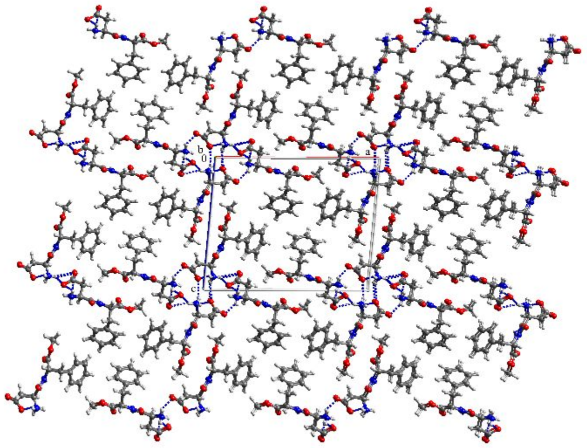

Figure S13. Crystal structure of aspartame

Table S11. Intermolecular energies (kJ mol^{-1}) calculated for KETXIR using PIXEL

dist.	Coul.	pol.	disp.	rep.	Pixel	Symm Op	
9.187	-113.2	-35.5	-21.6	38.6	-131.6	1-x,1/2+y,2-z	Intra stack H-bond
9.187	-113.2	-35.5	-21.6	38.6	-131.6	1-x,-1/2+y,2-z	Intra stack H-bond
10.545	-108.5	-37.2	-18.1	57.5	-106.3	-1+x,-1+y,+z	Intra stack H-bond
10.545	-108.5	-37.2	-18.1	57.5	-106.3	1+x,1+y,+z	Intra stack H-bond
4.96	-73.5	-34.3	-61.4	64.4	-104.8	+x,1+y,+z	Intra stack H-bond
4.961	-73.5	-34.3	-61.4	64.4	-104.8	+x,-1+y,+z	Intra stack H-bond
4.961	-70.6	-33.6	-59.9	66.8	-97.3	+x,1+y,+z	Intra stack H-bond
4.96	-70.6	-33.6	-59.9	66.8	-97.3	+x,-1+y,+z	Intra stack H-bond

3-Isobutyl-1-methylxanthine, CSD CEWVIJ10

The crystal structure of 3-isobutyl-1-methylxanthine is shown in Figure S11 and the intermolecular energies are in Table S14. The structure contains H-bonded dimers which are stacked along the short *a* axis. All of the interactions listed in Table 12 are intra stack interactions except the 8.574 Å interaction which is between adjacent stacks.

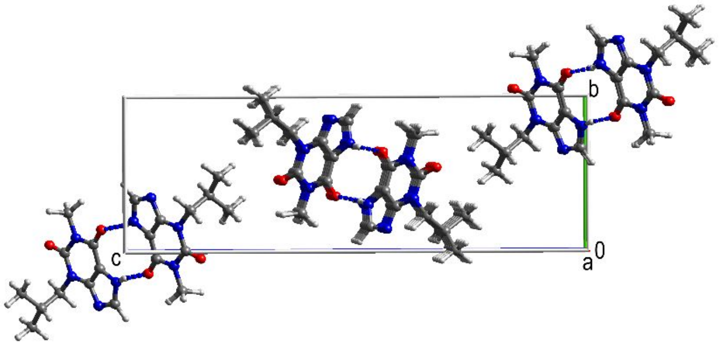

Figure S11. Crystal structure of 3-isobutyl-1-methylxanthine

**Table S14.** Intermolecular energies (kJ mol^{-1}) calculated for CEWVIJ10 using PIXEL							
dist.	Coul.	pol.	disp.	rep.	Pixel	Symm ops	
8.36	-94.7	-34.4	-22.6	71.7	-80	1-x,-y,-z	Dimer H-bond
4.882	-9.2	-6.2	-45	28.4	-32	1+x,+y,+z	Intra stack along *a*
4.882	-9.2	-6.2	-45	28.4	-32	-1+x,+y,+z	Intra stack along *a*
8.574	-18.9	-6.3	-14	16.5	-22.8	-x,-1-y,-z	Inter stack contact
6.183	-2.6	-4	-30	16.1	-20.5	-x,-y,-z	Inter stack contact

D-mannitol CSD DMANTL01

The crystal structure of D-mannitol is shown in Figure S12 and intermolecular energies are in Table S15. It is the very strong triple H-bond, 4.893 Å, which drives needle growth along the *c* axis

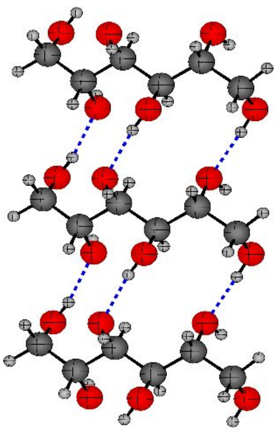

Figure S12. H-bonding in the crystal structure of D-mannitol

**Table S15.** Intermolecular energies (kJ mol^{-1}) calculated for DMANTL01 using PIXEL							
dist.	Coul.	pol.	disp.	rep.	Pixel	Symm Op	
4.893	-119.2	-47	-45.2	111.6	-99.9	+x,+y,1+z	H-bond chain along *c*
4.893	-119.2	-47	-45.2	111.6	-99.9	+x,+y,-1+z	H-bond chain along *c*
5.753	-53	-18.8	-34.8	60.1	-46.6	1/2+x,1/2-y,-z	Inter chain contact
5.753	-53	-18.8	-34.8	60.2	-46.5	-1/2+x,1/2-y,-z	Inter chain contact
8.227	-21.9	-6.3	-9.9	19.3	-18.8	3/2-x,-y,1/2+z	Inter chain contact
8.227	-21.9	-6.3	-9.9	19.3	-18.8	3/2-x,-y,-1/2+z	Inter chain contact

m-Nitroaniline, MNA, CSD MNIANL05

The interactions in the crystal structure of MNA are shown in Figure S13 and the intermolecular energies are in Table S16. The strongest interaction in the structure, -25.1 kJ/mol, is a 1D H-bond parallel to the *bc* diagonal, Figure S13, which does not influence crystal growth. It is the weaker stacking interaction along *c* (5.068 Å) which drives needle growth.

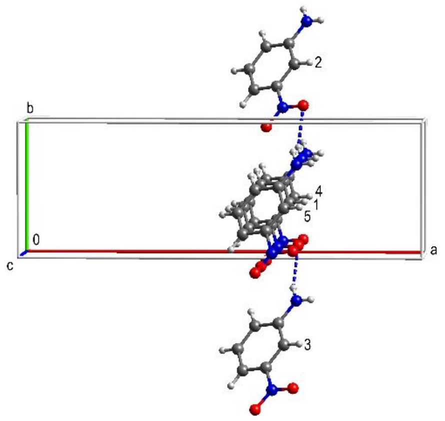

Figure S13. H-bonding in the crystal structure of MNA. There is a H-bond between molecules 3, 1 and 2 and molecules 5, 1 and 4 are stacked

**Table S16.** Intermolecular energies (kJ mol^{-1}) calculated for MNIANL05 using PIXEL							
dist.	Coul.	pol.	disp.	rep.	Pixel	Symm Op	
8.244	-21	-5.4	-11.8	13.1	-25.1	+x,1+y,-1+z	1D H-bond along *c*
8.244	-21	-5.4	-11.8	13.1	-25.1	+x,-1+y,1+z	1D H-bond along *c*
5.068	0.8	-1.6	-15.8	5.9	-10.7	+x,+y,1+z	Intra stack contact
5.068	0.8	-1.6	-15.8	5.9	-10.7	+x,+y,-1+z	Intra stack contact

4-Nitro-4'-methyl benzylidene aniline, NMBA, CSD NMBYAN01

The structure of NMBA is shown in Figure S14 and the intermolecular energies are in Table S17. The strongest interaction, 8.244 Å, is between the stacked molecules down the a axis and it involves C-H…O H-bonding. The stacking interaction down the a axis, 7.305 Å, drives needle growth.

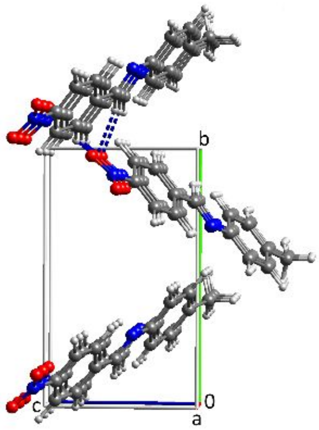

Figure S14. Stacking and C-H…O H-bonding in NMBA

**Table S17.** Intermolecular energies (kJ mol^{-1}) calculated for NMBYAN01 using PIXEL							
dist.	Coul.	pol.	disp.	rep.	Pixel	Symm Op	
8.373	-15.3	-6.9	-45.3	42.5	-25	1+x,+y,1+z	Intra stack along *c*
8.373	-15.3	-6.9	-45.3	42.5	-25	-1+x,+y,-1+z	Intra stack along *c*
7.305	-8.1	-3.6	-28.1	18.7	-21.2	1+x,+y,+z	Inter stack contact
7.305	-8.1	-3.6	-28.1	18.7	-21.2	-1+x,+y,+z	Inter stack contact

β-phthalocyanine, CSD PHTHCY14

The half molecule in the asymmetric unit was completed and the space group was reduced from $P2_1/c$ to $P2_1$.The crystal structure is shown in Figure S15 and intermolecular energies are in Table S18.

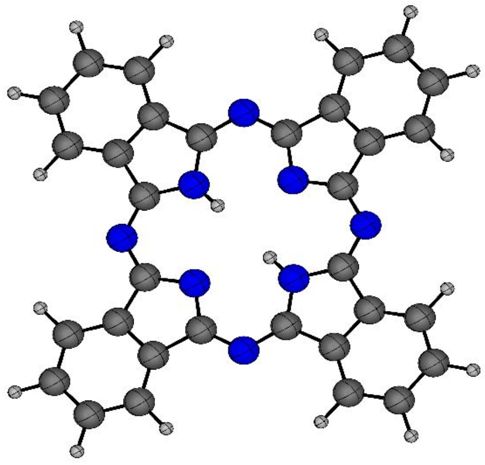

Figure S15. Crystal structure of β-phthalocyanine.

**Table S18.** Intermolecular energies (kJ mol^{-1}) for β-phthalocyanine calculated using PIXEL							
dist.	Coul.	pol.	disp.	rep.	Pixel	Symm Op	
4.707	-21.1	-11.7	-171	94.7	-109.1	x,1+y,z	Stack along *b*
10.068	-11.1	-5.4	-36.8	26.2	-27.1	1-x,1/2+y,1-z	Inter stack
10.068	-11.1	-5.4	-36.8	26.3	-27	-x,1/2+y,-z	Inter stack
12.832	-10.5	-4.6	-22.6	17.3	-20.4	1-x,1/2+y,-z	Inter stack
12.832	-10.5	-4.6	-22.6	17.3	-20.3	-x,-1/2+y,1-z	Inter stack

Lovastatin CSD CEKBEZ01

The crystal structure of lovastatin is shown in Figure S16 and intermolecular energies are in Table S17.

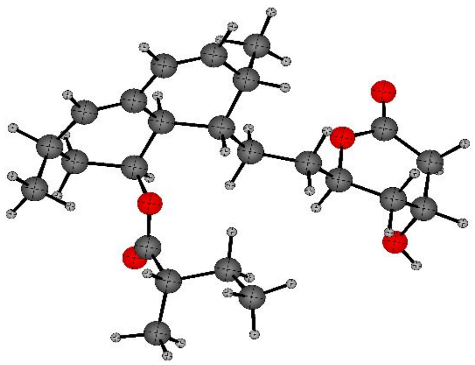

Figure S16. Crystal structure of lovastatin

**Table S19.** Intermolecular energies (kJ mol^{-1}) calculated for CEKBEZ01using PIXEL							
dist.	Coul.	pol.	disp.	rep.	Pixel	Symm Op	
5.89	-17.6	-11.2	-67.6	41.7	-54.8	1+x,+y,+z	vdW stack *a*
5.89	-17.6	-11.2	-67.6	41.7	-54.8	-1+x,+y,+z	vdW stack *a*
11.112	-26.4	-11.7	-20.3	26.9	-31.6	2-x,1/2+y,3/2-z	H-bond along *b*
11.112	-26.4	-11.7	-20.3	26.9	-31.6	2-x,-1/2+y,3/2-z	H-bond along *b*
11.393	-23.3	-7.7	-18.7	21	-28.7	1/2+x,3/2-y,1-z	Inter stack contact
11.393	-23.3	-7.7	-18.7	21	-28.7	-1/2+x,3/2-y,1-z	Inter stack contact
10.033	-9.3	-3.8	-32.4	23	-22.5	1/2+x,1/2-y,1-z	Inter stack contact
10.033	-9.3	-3.8	-32.4	23	-22.5	-1/2+x,1/2-y,1-z	Inter stack contact

Diflunisal form III CSD FAFWIS02

The crystal structure of of diflunisal form III is shown in Figure S17 and intermolecular energies are in Table S20.

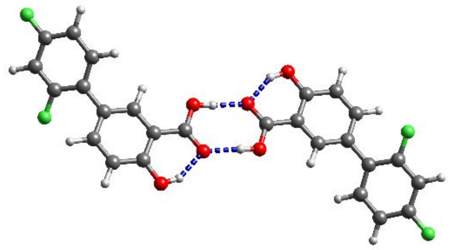

Figure S17. Crystal structure of diflunisal form III

The strongest interaction, 10.959 Å, is between the molecules in the H-bonded dimer. The dispersion dominated interaction, 3.836 Å, drives needle growth.

**Table S20.** Intermolecular energies (kJ mol^{-1}) calculated for FAFWIS02 using PIXEL							
dist.	Coul.	pol.	disp.	rep.	Pixel	Symm Op	
10.959	-114.3	-56	-21.7	123	-69.1		Dimer H-bond
3.836	-7.6	-5	-55.1	36.4	-31.3	+x,+y,1+z	Intra stack along *c*
3.836	-7.6	-5	-55.1	36.4	-31.3	+x,+y,-1+z	Intra stack along *c*
3.836	-8.9	-6	-57.5	42.6	-29.9	+x,+y,1+z	Intra stack along *c*
3.836	-8.9	-6	-57.5	42.6	-29.9	+x,+y,-1+z	Intra stack along *c*

References

(1) Küçükgüzel, S. G.; Mazi, A.; Sahin, F.; Öztürk, S.; Stables, J., Synthesis and biological activities of diflunisal hydrazide–hydrazones. *Eur. J. Med. Chem.* **2003,** *38*, 1005-1013.

(2) Vogt, F. G.; Yin, H.; Forcino, R. G.; Wu, L., 17O Solid-State NMR as a Sensitive Probe of Hydrogen Bonding in Crystalline and Amorphous Solid Forms of Diflunisal. *Mol. Pharm.* **2013,** *10*, 3433-3446.

(3) Uusi-Oukari, M.; Vähätalo, L.; Liljeblad, A., Modifications of Diflunisal and Meclofenamate Carboxyl Groups Affect Their Allosteric Effects on GABAA Receptor Ligand Binding. *Neurochem. Res.* **2014,** 39, 1183-1191.

(4) Laria, J. C. C. P.; Clauzel, L. M.; Olarte, A. Z.; Vicente, S. G.; Mian, A., Pharmaceutical Combinations Including Anti-Inflammatory and Antioxidant Conjugates Useful for Treating Metabolic Disorders; Google Patents: 2015.

(5) Li, Y.-l.; Qi, X.-y.; Jiang, H.; Deng, X.-d.; Dong, Y.-p.; Ding, T.-b.; Zhou, L.; Men, P.; Chu, Y.; Wang, R.-x.; Jiang, X.-c.; Ye, D.-y., Discovery, synthesis and biological evaluation of 2-(4-(N-phenethylsulfamoyl)phenoxy)acetamides (SAPAs) as novel sphingomyelin synthase 1 inhibitors. *Bioorg. Med. Chem.* **2015,** *23*, 6173-6184.

(6) Walshe, N.; Crushell, M.; Karpinska, J.; Erxleben, A.; McArdle, P., Anisotropic Crystal Growth in Flat and Nonflat Systems: The Important Influence of van der Waals Contact Molecular Stacking on Crystal Growth and Dissolution. *Crystal Growth and Design* **2015,** *15*, 3235-3248.

(7) Gbadebo, O. Ph.D. thesis, The investigation of methylmagnesium chloride as a non-nucleophilic base. National University of Ireland, Galway, Galway, 2019.

(8) Gbadebo, O.; Smith, D.; Harnett, G.; Donegan, G.; O'Leary, P., Surprising and Highly Efficient Use of Methylmagnesium Chloride as a Non-Nucleophilic Base in the Deprotonation and Alkylation of sp3 Centres Adjacent to Nitriles. *Eur. J. Org. Chem.* **2018,** *2018*, 7037-7045.

(9) McArdle, P., Oscail, a program package for small-molecule single-crystal crystallography with crystal morphology prediction and molecular modelling. *J. Appl. Crystallogr.* **2017,** *50*, 320-326.
